# Benchmarking AlphaFold‐enabled molecular docking predictions for antibiotic discovery

**DOI:** 10.15252/msb.202211081

**Published:** 2022-09-06

**Authors:** Felix Wong, Aarti Krishnan, Erica J Zheng, Hannes Stärk, Abigail L Manson, Ashlee M Earl, Tommi Jaakkola, James J Collins

**Affiliations:** ^1^ Institute for Medical Engineering & Science Massachusetts Institute of Technology Cambridge MA USA; ^2^ Department of Biological Engineering Massachusetts Institute of Technology Cambridge MA USA; ^3^ Infectious Disease and Microbiome Program Broad Institute of MIT and Harvard Cambridge MA USA; ^4^ Program in Chemical Biology Harvard University Cambridge MA USA; ^5^ Computer Science and Artificial Intelligence Laboratory Massachusetts Institute of Technology Cambridge MA USA; ^6^ Wyss Institute for Biologically Inspired Engineering Harvard University Boston MA USA

**Keywords:** AlphaFold2, enzymatic activity, machine learning, molecular docking, protein‐ligand interactions, Computational Biology, Pharmacology & Drug Discovery, Structural Biology

## Abstract

Efficient identification of drug mechanisms of action remains a challenge. Computational docking approaches have been widely used to predict drug binding targets; yet, such approaches depend on existing protein structures, and accurate structural predictions have only recently become available from AlphaFold2. Here, we combine AlphaFold2 with molecular docking simulations to predict protein‐ligand interactions between 296 proteins spanning *Escherichia coli*'s essential proteome, and 218 active antibacterial compounds and 100 inactive compounds, respectively, pointing to widespread compound and protein promiscuity. We benchmark model performance by measuring enzymatic activity for 12 essential proteins treated with each antibacterial compound. We confirm extensive promiscuity, but find that the average area under the receiver operating characteristic curve (auROC) is 0.48, indicating weak model performance. We demonstrate that rescoring of docking poses using machine learning‐based approaches improves model performance, resulting in average auROCs as large as 0.63, and that ensembles of rescoring functions improve prediction accuracy and the ratio of true‐positive rate to false‐positive rate. This work indicates that advances in modeling protein‐ligand interactions, particularly using machine learning‐based approaches, are needed to better harness AlphaFold2 for drug discovery.

## Graphical abstract

Assessing molecular docking simulations based on AlphaFold2‐predicted structures with high‐throughput measurements of protein‐ligand interactions reveals weak model performance. Machine learning‐based approaches improve performance and better harness AlphaFold2 for drug discovery.

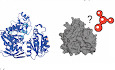

## Introduction

A major challenge in drug discovery is the identification of drug‐target interactions. Various approaches to identifying molecular drug targets have been developed, including those based on biochemical assays, genetic interactions, and molecular docking (Kitchen *et al*, 2004; Schenone *et al*, 2013). Molecular docking, in particular, has proven versatile for identifying protein‐ligand interactions and drug mechanisms of action. In molecular docking, ligand binding poses within a targeted binding site of a protein are computationally modeled using scoring functions, and poses are optimized to provide structural information and activity predictions in the form of thermodynamic binding affinities. While docking has been used to enrich for potential hit compounds that bind pre‐specified proteins in “one target, many compounds” approaches, the process of “reverse docking,” in which a small molecule is docked across different potential protein targets, leverages docking to discover binding partners and drug mechanisms of action (Kharkar *et al*, 2014; Lee *et al*, 2016). Although versatile, reverse docking requires *a priori* knowledge of the protein structures of interest, and its application to drug‐target identification has been limited by the number and quality of target protein structures (Chen & Zhi, 2001; Kharkar *et al*, 2014; Lee *et al*, 2016).

**Figure 1:**
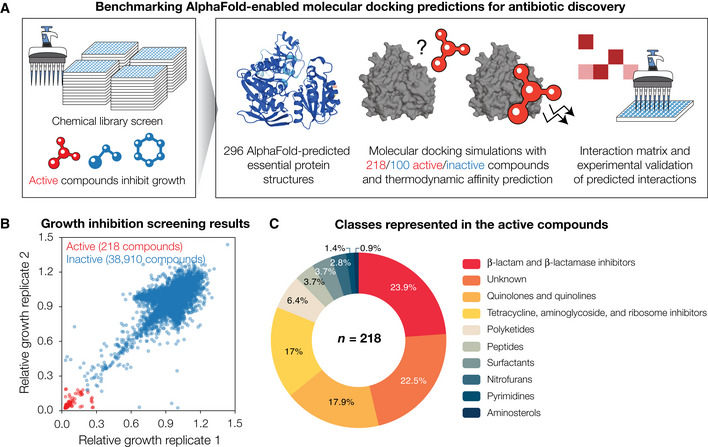
Growth inhibition screens in *Escherichia coli* reveal 218 active compounds, whose interactions with essential proteins are predicted by combining AlphaFold2 with molecular docking ASchematic of the approach. To define our chemical space of interest, we performed high‐throughput screens of growth inhibition against wild‐type *E. coli*. Compounds that inhibited growth were taken as active, and each active compound was computationally docked with each of 296 AlphaFold2‐predicted *E. coli* essential protein structures. For comparison, a subset of the inactive compounds was docked in the same way. An interaction matrix showing the thermodynamic binding affinities predicted by the docking simulations was then constructed. A protein‐ligand interaction was predicted to occur if its predicted binding affinity was smaller than a threshold value. All possible interactions for a subset of essential proteins, including those not predicted to occur, were empirically tested to benchmark model performance.BGrowth inhibition measurements for 39,128 compounds, from which 218 compounds (including known antibiotics) were identified as active against *E. coli* BW25113. Data are shown from two biological replicates. Compounds with mean relative growth less than 0.2 were classified as active (red points), and all other compounds were classified as inactive (blue points).CDistribution of the compound classes represented in the 218 active compounds.

Here, we reasoned that the recent release of the AlphaFold2 database of protein structure predictions (Jumper *et al*, 2021; Varadi *et al*, 2022) could enable reverse docking approaches that span *Escherichia coli*'s essential proteome, allowing for the extensive prediction of binding targets of antibacterial compounds (Fig 1A). We hypothesized that such an approach could enrich for true protein‐ligand interactions from the large, combinatorial space of all possible interactions between antibacterial compounds and essential proteins. As computational docking approaches are known to predict many false positives (Adeshina *et al*, 2020), the predicted protein‐ligand interactions could be experimentally interrogated, in part, using biochemical assays that measure enzymatic activity, with binding interactions supported by enzymatic inhibition. In addition to inspiring further studies that expand on the interactions discovered in this way, these experiments could be used to benchmark the performance of our modeling platform and reveal the prediction accuracy possible with AlphaFold2‐enabled molecular docking simulations.

To this end, we assembled a set of antibacterial compounds arising from a high‐throughput growth inhibition screen against *Escherichia coli*. We then deployed computational docking simulations using AutoDock Vina (Eberhardt *et al*, 2021) and AlphaFold2‐predicted protein structures to identify protein‐ligand interactions between these antibacterial compounds and all proteins from *E. coli*'s essential proteome. These simulations predicted both specific protein‐ligand interactions and widespread compound and protein promiscuity. By assembling a set of known or inferred antibiotic binding interactions from the literature, we found that our predictions only partially recapitulate these interactions. To further test our predictions, we measured enzymatic activity for diverse essential *E. coli* proteins involved in DNA replication, transcription, metabolism, and cell wall synthesis. Treatment of each protein with each antibacterial compound revealed that multiple compounds inhibit enzymatic activity, confirming extensive promiscuity and enabling statistical benchmarking of model performance. Detailed comparisons of our *in silico* predictions with experimental data showed that our approach predicted empirical protein‐ligand interactions with an average accuracy between 41 and 73%, depending on the binding affinity threshold used. Independent of the binding affinity threshold, the area under the receiver operating characteristic curve (auROC) across the essential proteins tested ranged from 0.18 to 0.71 (average 0.48). Furthermore, model performance was similar using experimentally determined protein structures. In view of the observation that a random model corresponds to an auROC of 0.5, these findings indicate that molecular docking simulations exhibit weak performance.

Computational docking platforms based on different scoring functions are widely available. Notably, machine learning‐based scoring functions have previously been shown to improve docking performance, as measured by the auROC (Ballester & Mitchell, 2010; Durrant & McCammon, 2010; Pereira *et al*, 2016; Wójcikowski *et al*, 2017, 2019). To assess the robustness of our results to variation in the docking methods used, we considered alternative docking approaches involving another docking platform (DOCK6.9; Allen *et al*, 2015) and machine learning‐based scoring functions. By rescoring our predictions with four machine learning‐based scoring functions—RF‐Score (Ballester & Mitchell, 2010), RF‐Score‐VS (Wójcikowski *et al*, 2017), PLEC score (Wójcikowski *et al*, 2019), and NNScore (Durrant & McCammon, 2010)—we found improvements in performance, as measured by the auROC, with three of the four scoring functions (RF‐Score, RF‐Score‐VS, and NNScore). In contrast, employing DOCK6.9 and rescoring with the PLEC score did not improve model performance. Lastly, we show that consensus models comprising several machine learning‐based scoring functions improve prediction accuracy and the ratio of true‐positive rate to false‐positive rate. Taken together, these results demonstrate the need to further develop methods of more accurately modeling protein‐ligand interactions and suggest the potential of machine learning to improve modeling predictions. By providing a comprehensive dataset for benchmarking protein‐ligand interaction predictions and demonstrating how machine learning can better harness AlphaFold2‐predicted protein structures for molecular docking, our work informs the application of AlphaFold2 to drug discovery.

## Results

### A screen of 39,128 compounds reveals 218 antibacterial compounds active against *Escherichia coli*

We first defined our chemical space of interest by screening a library of 39,128 unique compounds comprising the most clinically used antibiotics, natural products, and structurally diverse molecules with molecular weights between 40 Da and 4,200 Da—a range which includes those of most known antibiotics—for growth inhibition against wild‐type *E. coli* K‐12 BW25113 (Dataset EV1). Compounds were screened at 50 μM with cells grown in LB medium, and optical density values after overnight incubation were measured. Defining active compounds as those that inhibit relative growth by 80%, we found 218 structurally diverse compounds with activity (Fig 1B). Most (∼ 80%) of the 218 active compounds could be classified into known antibiotic structural classes, including the β‐lactam, aminoglycoside, tetracycline, quinolone, and polyketide classes (Fig 1C). The remaining active compounds comprised of known antibacterial compounds—including toxins and antineoplastic compounds—and additional compounds whose antibacterial activities against *E. coli* have not previously been reported (Dataset EV1).

### Molecular docking of compounds with AlphaFold2‐predicted *Escherichia coli* essential protein structures

**Figure 2:**
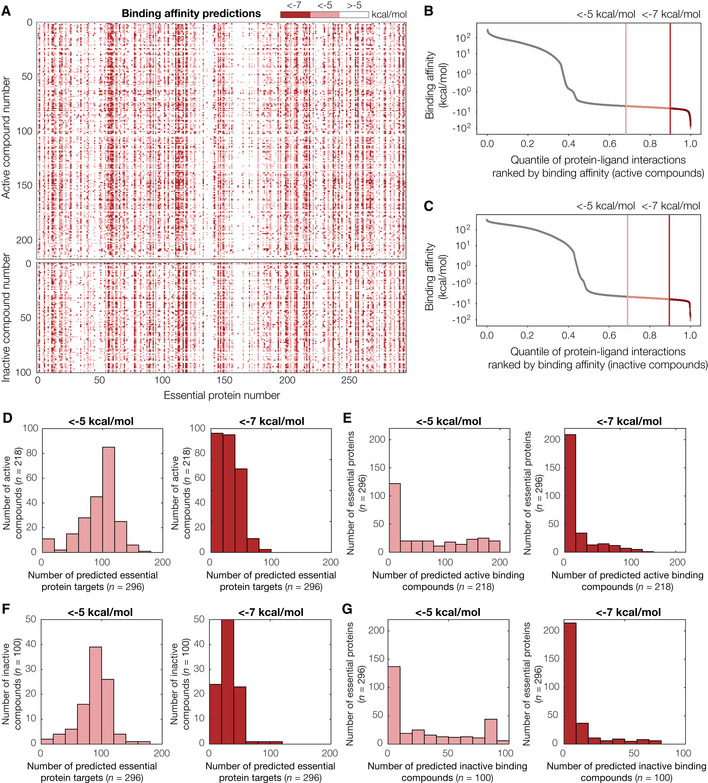
Binding affinity predictions for 218 active compounds, 100 inactive compounds, and 296 AlphaFold2‐predicted *Escherichia coli* essential protein structures AInteraction matrix showing the predicted binding affinities (kcal/mol) between all pairs of active or inactive compounds and essential proteins modeled, discretized into bins of < −7 kcal/mol (strong predicted binding), < −5 kcal/mol (moderate predicted binding), and > −5 kcal/mol (no predicted binding). Predictions for active compounds are shown at top, and inactive compounds are shown at bottom.B, CRank‐ordered binding affinities for the protein‐ligand pairs modeled by our approach. Vertical lines indicate binding affinity thresholds of −5 kcal/mol and −7 kcal/mol. Plots are for protein‐ligand interactions involving all 218 active compounds (B) or 100 inactive compounds (C).DHistograms of numbers of predicted essential protein targets with binding affinity < −5 kcal/mol (left) or < −7 kcal/mol (right), for all 218 active compounds.EHistograms of numbers of predicted binding compounds with binding affinity < −5 kcal/mol (left) or < −7 kcal/mol (right), for all 296 essential proteins.F, GSimilar to (D–E), but for all 100 inactive compounds modeled.

**Figure EV1:**
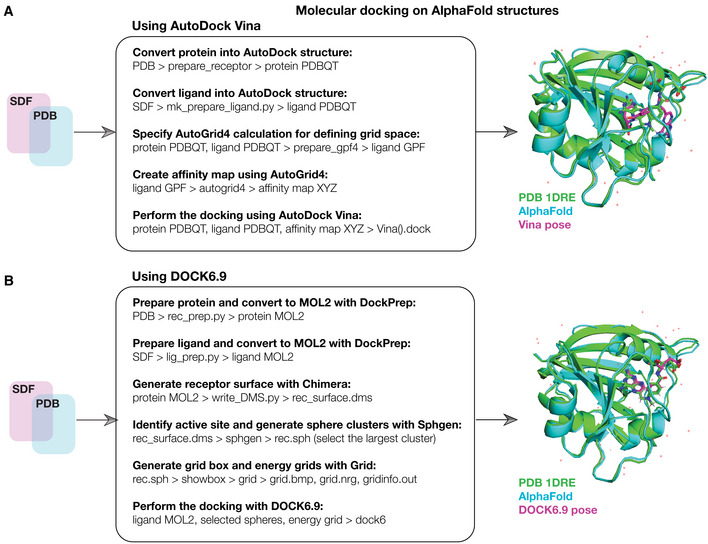
Schematic of the computational docking approach A(Left) Schematic of the computational docking approach using AutoDock Vina. 296 essential proteins in *E. coli* were identified, and their AlphaFold2‐predicted structures were curated. The 218 active compounds and 100 inactive compounds were represented in three dimensions in SDF files. All compounds and proteins were prepared for docking as shown and then docked using AutoDock Vina run on a high‐performance computing server. The resulting binding pose and thermodynamic binding affinity predictions for all 64,528 (active compounds) and 29,600 (inactive compounds) pairwise protein‐ligand interactions were analyzed and ranked. (Right) Superimposed predicted and experimental structures for methotrexate binding to *E. coli* FolA (PDB 1DRE), which was used as a positive docking control from the Protein Data Bank (Dataset EV2).BSimilar to (A), but for the docking of 218 active compounds and 100 inactive compounds, and a subset of 12 essential proteins, respectively, using DOCK6.9. The 12 selected essential proteins correspond to all proteins empirically tested in this study.

We next investigated the potential binding targets of all active compounds, as predicted by molecular docking with AlphaFold2‐predicted protein structures. We reasoned that many active compounds exert their antibacterial activities largely by interacting with essential proteins in *E. coli*. Previous studies have identified essential genes in *E. coli* using transposon‐directed insertion site sequencing (Goodall *et al*, 2018) and CRISPR interference screening (Rousset *et al*, 2018, 2021). Building on these studies, we shortlisted genes identified as essential in at least two of the three studies, resulting in a total of 296 out of ∼ 4,000 total genes in *E. coli* (Blattner *et al*, 1997; Materials and Methods and Dataset EV2). As positive controls for our docking simulations, we additionally included experimentally determined structures in complex with various ligands from the Protein Data Bank (Berman *et al*, 2000; Dataset EV2). We proceeded to dock all 218 active compounds against the 296 AlphaFold2‐predicted essential protein structures using AutoDock Vina, a widely used and benchmarked open‐source program for docking (Pereira *et al*, 2016; Vieira & Sousa, 2019; Eberhardt *et al*, 2021; Fig EV1). We describe and compare our approach with different docking methods and introduce relevant concepts, in Box 1. In total, our approach resulted in binding pose and binding affinity predictions for 64,528 protein‐ligand pairs (Fig 2A and Dataset EV2). For comparison, we performed analogous docking simulations for 100 randomly selected inactive compounds, which resulted in binding pose and affinity predictions for 29,600 protein‐ligand pairs (Fig 2A and Dataset EV2).

Box 1 **Integrating AlphaFold2 with molecular docking.**

Different software for performing molecular docking are widely available and commonly used platforms include AutoDock Vina (Eberhardt *et al*, 2021) and DOCK (Allen *et al*, 2015). Docking aims to estimate the binding pose of a ligand interacting with a macromolecule, such as a protein, and associated quantities such as the binding affinity. How this is done depends on the software used: some platforms, such as AutoDock Vina, rely on empirical free energy scoring functions that aim to directly estimate the free energy of binding for a pose, while others such as DOCK use force field‐based scoring functions that account for intermolecular van der Waals and electrostatic interactions between the protein and ligand. Recent advances in integrating machine learning with docking have resulted in machine learning‐based scoring functions, and their use to rescore poses generated by other docking platforms (Ballester & Mitchell, 2010; Durrant & McCammon, 2010; Pereira *et al*, 2016; Wójcikowski *et al*, 2017, 2019).

As shown in the workflow here, in order to leverage AlphaFold2 for docking, we first downloaded all 296 AlphaFold2‐predicted *E. coli* essential protein structures from the AlphaFold Protein Structure Database (Jumper *et al*, 2021; Varadi *et al*, 2022). We assembled a list of simplified molecular‐input line‐entry system (SMILES) strings describing the chemical structures of our 218 antibacterial compounds of interest and prepared the compounds and proteins for docking as required for the program used. As a key input to docking, the active site of each protein must be specified. Blind docking approaches computationally estimate active sites; alternatively, active sites can be specified based on those empirically evidenced in the Protein Data Bank. As the active sites for all protein structures were not known, we used blind docking to identify potential active sites and supplemented the active site selection with information from the Protein Data Bank (when available) for our assessments of model performance. We used AutoDock Vina to predict binding poses and binding affinities for all protein‐ligand pairs of interest. The resulting binding affinities (kcal/mol) can be interpreted as the free energy of ligand binding, with lower energies indicating stronger binding. Analogous binding affinities from DOCK6.9 are represented by grid scores (kcal/mol), which measure binding energy but should not be directly compared with the free energies predicted by AutoDock Vina. Binding affinities predicted by the machine learning‐based rescoring functions considered in this work are represented by *pK*_*d*_ values—equal to the negative logarithm of the dissociation constant—and higher *pK*_*d*_ values indicate stronger binding.

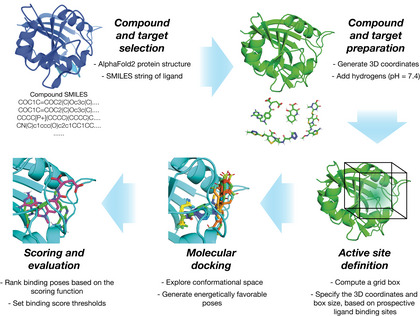

Upon analyzing the predicted binding affinities, we found that our approach predicted widespread compound and protein promiscuity for both active and inactive compounds. For a stringent binding affinity threshold of −7 (−5) kcal/mol—corresponding to the highest‐ranked 9.6% (31%) of the predicted binding affinities (Fig 2B)—we found that, of the 218 active compounds screened, 187 (207) were predicted to bind to at least three proteins (Fig 2D). Additionally, of the 296 essential proteins screened, 178 (216) were predicted to bind to at least three compounds (Fig 2E). Similar binding affinity thresholds apply to the 100 inactive compounds screened (Fig 2C), of which 86 (99) were predicted to bind at least three proteins (Fig 2F), and 137 (204) essential proteins were predicted to bind to at least three compounds (Fig 2G). These findings suggest that docking does not distinguish between active and inactive compounds and point to potential limitations in docking performance. Nevertheless, as molecular docking is known to produce many false positives (Adeshina *et al*, 2020; Bender *et al*, 2021), we further investigated the performance of our approach by (i) comparing its predictions with known antibiotic binding targets and (ii) experimentally interrogating the predicted protein‐ligand interactions involving active compounds, as described below.

### Comparing model predictions with known antibiotic binding targets

We first assessed the performance of our approach by comparing its predictions to known interactions involving commonly used classes of antibiotics. We searched the literature for previously studied antibiotic‐protein target pairs (as described in detail in Materials and Methods) and assembled a dataset comprising 142 experimentally evidenced or inferred interactions in *E. coli* (Dataset EV3). The compounds in this dataset represent diverse antibiotic classes and target various proteins, such as the 30S ribosomal subunit and the enoyl‐acyl carrier protein reductase FabI. Of the 142 curated antibiotic‐protein interactions, we found that the model correctly predicted only 3 interactions with a binding affinity threshold of −7 kcal/mol and 43 interactions with a binding affinity threshold of −5 kcal/mol, resulting in true‐positive rates of 2.1 and 30.3%, respectively. While an assessment of the false‐positive rate with this data may have limitations—the lack of evidence of an antibiotic‐protein interaction does not necessarily imply that there is no such interaction—the same binding affinity thresholds encompass 9.6% (−7 kcal/mol) and 31% (−5 kcal/mol) of the modeled protein‐ligand interactions involving active compounds, as described above. If true protein‐ligand interactions were rare, this would suggest that the false‐positive rates predicted by our model are comparable to its true‐positive rates, even for a stringent binding affinity threshold of −7 kcal/mol. Consistent with this reasoning, the same binding affinity thresholds encompass 10% (−7 kcal/mol) and 30% (−5 kcal/mol) of the modeled protein‐ligand interactions involving inactive compounds (Fig 2C), which are likely to not bind any essential protein given that they do not inhibit bacterial growth. This comparison, therefore, suggests that the performance of our modeling platform is weak. Although various thresholds may be chosen to reflect one's desired stringency, based on these results we assumed −7 kcal/mol to be a stringent binding affinity threshold, and −5 kcal/mol to be an inclusive binding affinity threshold. We further compare the results with both thresholds for our assessments of model performance below.

### Enzymatic inhibition measurements for 12 essential proteins reveal widespread promiscuity

**Figure 3:**
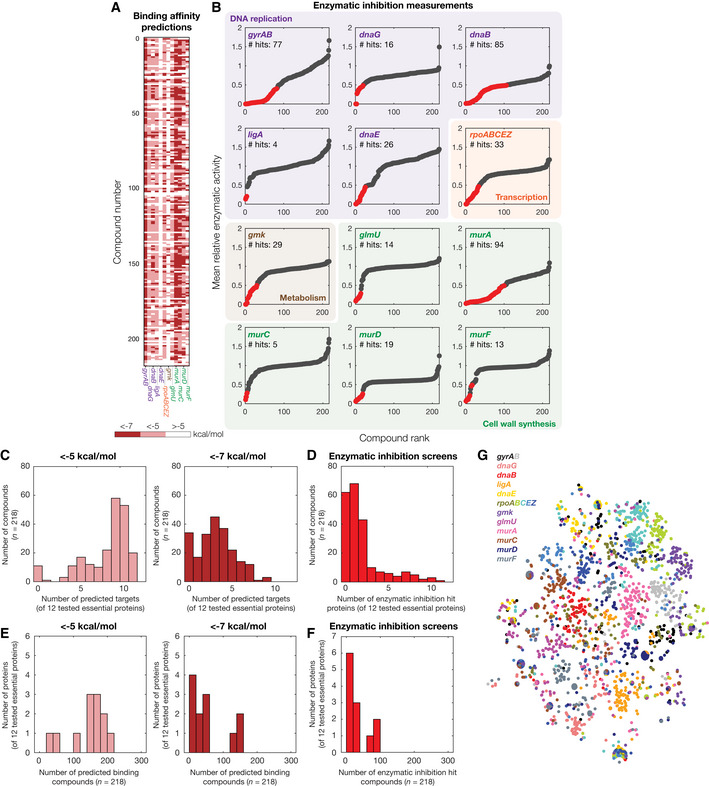
*In vitro* enzymatic measurements of protein‐ligand interactions AInteraction matrix showing the predicted binding affinities (kcal/mol) between all pairs of active compounds and 12 essential proteins tested for enzymatic inhibition, discretized into bins of < −7 kcal/mol, < −5 kcal/mol, and > −5 kcal/mol.BRank‐ordered mean relative enzymatic activity across all 218 active compounds, at a final concentration of 100 μM, for each of 12 essential proteins experimentally tested for enzymatic inhibition. Essential proteins correspond to the genes indicated and are involved in DNA replication (purple), transcription (orange), metabolism (brown), and cell wall synthesis (green). Results show the mean of two biological replicates, and relative activity is measured with respect to untreated controls. Binding interaction hits are protein‐ligand interactions with relative enzymatic activities of < 50% in both replicates (red points). All other interactions are designated as non‐hits (gray points).CHistogram of numbers of predicted essential protein targets with binding affinity < −5 kcal/mol (left) or < −7 kcal/mol (right), for all 218 active compounds and all 12 essential proteins tested in (B).DHistogram of numbers of enzymatic inhibition hit proteins, for all 218 active compounds and all 12 essential proteins tested in (B).EHistogram of numbers of predicted binding compounds with binding affinity < −5 kcal/mol (left) or < −7 kcal/mol (right), for all 218 active compounds and all 12 essential proteins tested in (B).FHistogram of numbers of enzymatic inhibition hit compounds, for all 218 active compounds and all 12 essential proteins tested in (B).Gt‐SNE plot of protein‐ligand interaction fingerprints, colored by protein and protein subunit.

**Figure EV2:**
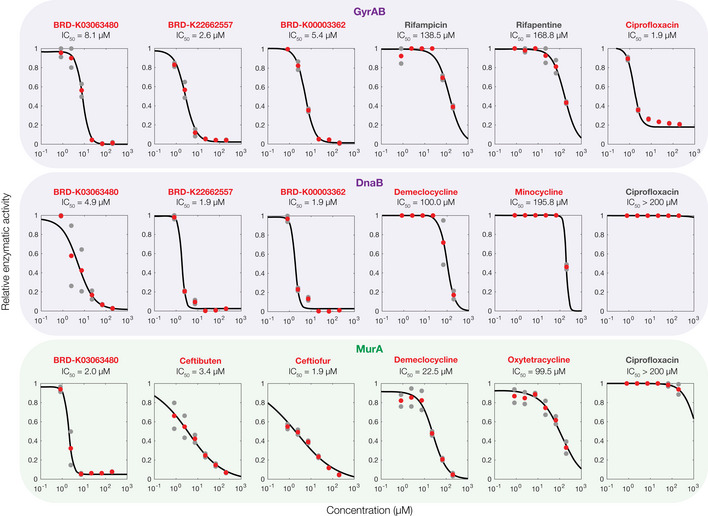
Enzymatic inhibition screen validation experiments Dose–response experiments showing the relative enzymatic activity of three essential proteins, each treated with six antibacterial compounds. Relative activity is measured with respect to untreated controls. Data from two biological replicates (gray points) are shown, and mean activity values (red points) were fit to Hill functions (black curves) to estimate IC_50_ values. Compound names in red indicate hits from the screens shown in Fig 3 of the main text, and compound names in gray indicate non‐hits. Essential proteins correspond to the genes indicated and are involved in DNA replication (purple) and cell wall synthesis (green).

Given that our approach generated essential proteome‐wide predictions of protein‐ligand binding, we aimed to further test a subset of these predictions experimentally. We reasoned that many predictions could be validated or refuted using *in vitro* enzymatic assays, in which proteins with enzymatic activity are reconstituted and ligand binding is assessed by measuring enzymatic inhibition. We considered a panel of 12 essential *E. coli* proteins or protein complexes for which enzymatic assays were available, including DNA gyrase (*gyrAB*), DNA primase (*dnaG*), DNA helicase (*dnaB*), NAD^+^‐dependent DNA ligase (*ligA*), DNA polymerase III subunit α (*dnaE*), RNA polymerase (*rpoABCEZ*), guanylate kinase (*gmk*), GlmU (a bifunctional acetyltransferase), MurA, MurC, MurD, and MurF (peptidoglycan cell wall synthases; Fig 3A). These proteins are diverse and participate in various cellular processes including DNA replication, transcription, metabolism, and cell wall synthesis (Fig 3B). We screened all 218 active compounds for enzymatic inhibition against this panel in duplicate at a concentration of 100 μM. Building on studies indicating that compounds with half‐maximal inhibitory concentrations (IC_50_) ≤ 50 μM are low enough to be lead‐like (McLay, 2003), we chose the concentration of 100 μM to be high enough such that hits possess at least mild inhibitory activity *in vitro*. We then classified binding interaction hits as those for which the enzymatic activity was < 50% of that of untreated controls in both replicates. Across all proteins, we found that widespread inhibition of enzymatic activity occurred for treatment with various compounds (Fig 3B and Dataset EV4). MurA and DNA helicase displayed the largest numbers of binding interaction hits, with 94 and 85 hits, respectively; in contrast, we found only 4 hits for DNA ligase, and 5 hits for MurC, which were the proteins with the fewest numbers of hits (Fig 3B). To improve reproducibility, we performed dose–response measurements of a small subset of our initial screening hits and non‐hits, which, after curve‐fitting, revealed IC_50_ values between 1.9 and 195.8 μM for hits, and > 100 μM for non‐hits (Fig EV2). These findings suggest that our binding interaction hits encapsulate a range of binding affinities from strong (micromolar) to weak (hundred‐micromolar). However, it is important to note that, as many antibiotic binding interactions have IC_50_ values in the (sub‐)micromolar range (Khodursky *et al*, 1995; Kocaoglu & Carlson, 2015), it is possible that only strong binding affinities are relevant to antibacterial action.

Intriguingly, upon statistically analyzing our enzymatic inhibition screens, we found that 45 compounds promiscuously inhibited at least three tested proteins. Additionally, as mentioned above, all tested essential proteins were inhibited by at least four distinct compounds. Although we observed differences between the predicted and experimentally observed binding interactions (as assessed below), this observation is consistent with the wide ranges of protein target and binding compound numbers predicted by our docking, and the shapes of the empirical distributions are better captured by docking using more stringent binding affinity thresholds (Fig 3C–F). To better understand whether the widespread promiscuity predicted by our docking simulations arises from shared attributes in protein‐ligand interactions, we used t‐distributed stochastic neighborhood embedding (t‐SNE) to visualize the protein‐ligand interaction fingerprint of each docked pose across all empirically tested proteins (Fig 3G). Here, points that are closer in distance represent structurally similar protein‐ligand interaction fingerprints. This visualization showed that the modeled protein‐ligand interactions formed few large clusters, suggesting that the predicted promiscuity arises in a protein‐ and ligand‐specific manner from our docking simulations. Taken together, these results suggest that promiscuity is an emergent, non‐trivial feature of our docking predictions that is consistent with enzymatic inhibition measurements. Moreover, our enzymatic inhibition measurements provide empirical data to directly benchmark the prediction accuracy of our approach.

### Benchmarking model performance

**Figure 4:**
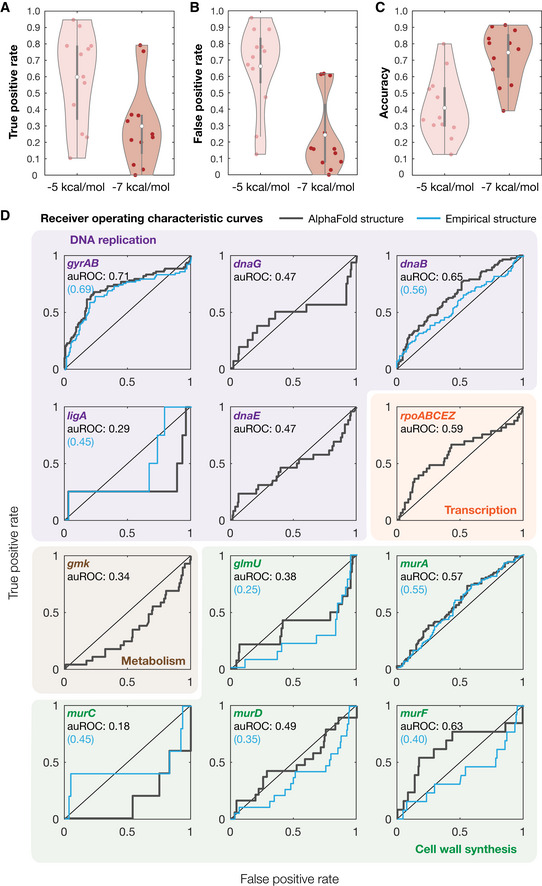
Benchmarking model performance A–CDistributions of true‐positive rates (A), false‐positive rates (B), and accuracy (C) across all 12 empirically tested essential proteins, for binding affinity thresholds of −5 kcal/mol and −7 kcal/mol. White points indicate mean values, and gray bars indicate ranges of 25^th^ to 75^th^ percentile values (*Q*_1_ and *Q*_3_, respectively). The whiskers of the gray box plots indicate ranges of values not considered outliers, that is, those between *Q*_1_ – 1.5 × IQR and *Q*_3_ + 1.5 × IQR, where IQR = *Q*_3_ – *Q*_1_ is the interquartile range.DReceiver operating characteristic (ROC) curves (gray) for all 12 empirically tested essential proteins. Essential proteins correspond to the genes indicated and are involved in DNA replication (purple), transcription (orange), metabolism (brown), and cell wall synthesis (green). The black diagonal line indicates the benchmark generated by random guessing. Blue curves are ROC curves generated using experimentally determined protein structures, where available. auROC—area under the ROC curve.

We next sought to statistically benchmark the performance of our modeling platform. Building on our enzymatic inhibition measurements, we compared the experimentally observed binding interaction hits against our predicted interactions with binding affinities less than −5 kcal/mol and −7 kcal/mol (Fig 4A–C). This comparison revealed that the true‐positive rates of our approach, averaged across all 12 essential proteins tested, were 59% (−5 kcal/mol threshold) and 30% (−7 kcal/mol threshold), respectively (Fig 4A). Average false‐positive rates were similar, with values of 66 and 24%, respectively (Fig 4B), while the average accuracy was 41% (−5 kcal/mol threshold) and 73% (−7 kcal/mol threshold), respectively (Fig 4C). As expected, more stringent binding affinity thresholds result in less binding interaction predictions and are associated with lower true‐positive rates and higher accuracy. Nevertheless, as a random model would, on average, exhibit true‐positive rates equal to false‐positive rates, our approach only performs better‐than‐random (on average) for the more stringent binding affinity threshold of −7 kcal/mol. Indeed, our approach performs better‐than‐random for only 5 (*dnaB*, *dnaE*, *rpoABCEZ*, *murA*, and *murF*) of the 12 essential proteins tested for the inclusive binding affinity threshold of −5 kcal/mol and for 9 essential proteins (*gyrAB*, *dnaG*, *dnaB*, *ligA*, *rpoABCEZ*, *glmU*, *murA*, *murD*, and *murF*) for the stringent binding affinity threshold of −7 kcal/mol (Fig 4A and B). These results indicate that model performance can vary from being weak to moderate depending on the binding affinity threshold used.

**Figure EV3:**
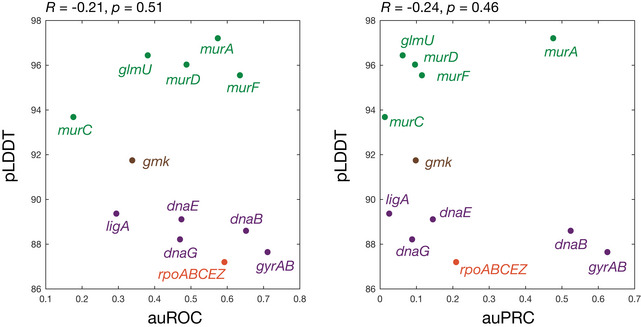
No correlation between model performance and AlphaFold2 prediction confidence Shown is a plot of the auROC values from Fig 4D of the main text or auPRC values from Fig EV5 against AlphaFold2's per‐residue confidence score (pLDDT), averaged across each protein, for all 12 empirically tested essential proteins. Essential proteins correspond to the genes indicated and are involved in DNA replication (purple), transcription (orange), metabolism (brown), and cell wall synthesis (green). Higher pLDDT scores indicate higher model confidence. The Pearson correlation coefficients, *R*, and corresponding *P*‐values are indicated.

**Figure EV4:**
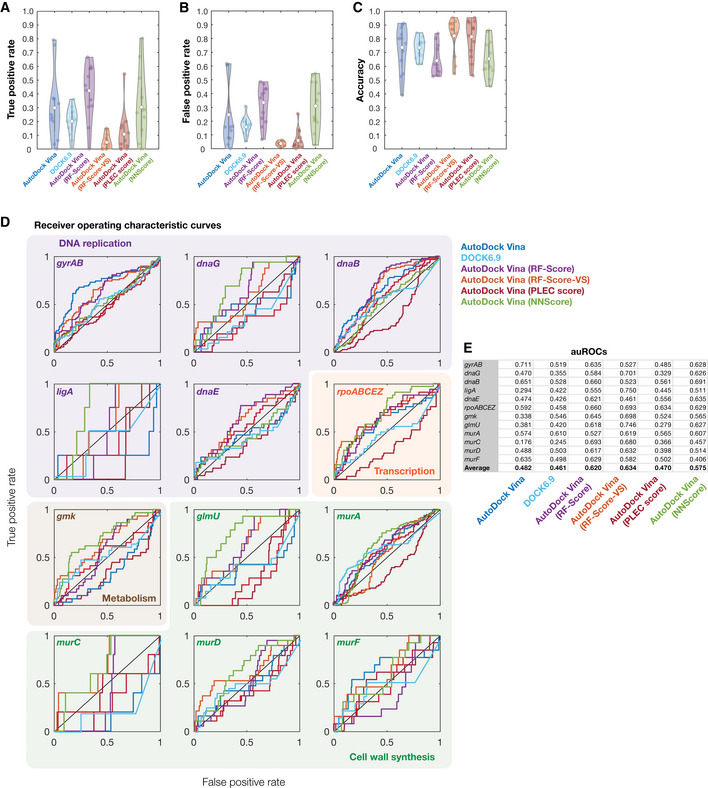
Model performance for different rescoring functions A–CDistributions of true‐positive rates (A), false‐positive rates (B), and accuracy (C) across all 12 empirically tested essential proteins, for binding affinity thresholds of −7 kcal/mol (AutoDock Vina), −70 kcal/mol (DOCK6.9), or *pK*_*d*_ > 7 (AutoDock Vina with all rescoring functions). White points indicate mean values, and gray bars indicate ranges of 25^th^ to 75^th^ percentile values (*Q*_1_ and *Q*_3_, respectively). The whiskers of the gray box plots indicate ranges of values not considered outliers, that is, those between *Q*_1_ – 1.5 × IQR and *Q*_3_ + 1.5 × IQR, where IQR = *Q*_3_ – *Q*_1_ is the interquartile range.DReceiver operating characteristic (ROC) curves for all 12 empirically tested essential proteins. Essential proteins correspond to the genes indicated and are involved in DNA replication (purple), transcription (orange), metabolism (brown), and cell wall synthesis (green). The black diagonal line indicates the benchmark generated by random guessing. ROC curves are colored according to the model used. Curves for AutoDock Vina (dark blue curves) are identical to those shown in Fig 4D of the main text and are reproduced here to facilitate comparison between models.EArea under the ROC curve (auROC) values for each empirically tested essential protein and each model used.

**Figure EV5:**
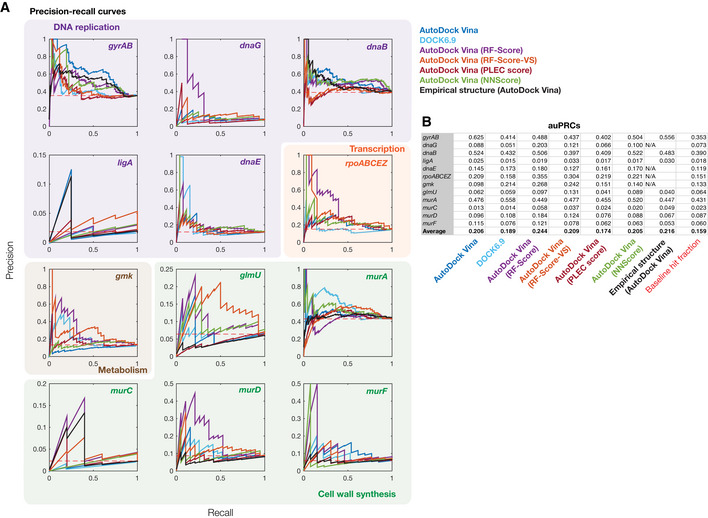
Precision‐recall for different rescoring functions APrecision‐recall (PR) curves for all 12 empirically tested essential proteins. Essential proteins correspond to the genes indicated and are involved in DNA replication (purple), transcription (orange), metabolism (brown), and cell wall synthesis (green). The red dashed line indicates the benchmark generated by random guessing, corresponding to recall values equal to the baseline hit fraction. PR curves are colored according to the model or protein structure used.BArea under the PR curve (auPRC) values for each empirically tested essential protein and each model used.

It is convenient to assess the performance of our approach independently of binding affinity thresholds, a task for which receiver operating characteristic (ROC) curves are well suited. The ROC curve of a model plots the false‐positive rate against the true‐positive rate, with the diagonal indicating the performance expected from a random model. The area under the ROC curve (auROC) can be interpreted as the probability of correctly classifying a pair of samples (e.g., a binding interaction hit and non‐hit). We found that the auROC values across all 12 essential proteins tested ranged from 0.18 (*murC*) to 0.71 (*gyrAB*), with an average value of 0.48 (Fig 4D). This assessment suggests that this approach performs, on average, marginally worse than random (auROC = 0.50), and further indicates that model performance can vary from being weak to moderate depending on the protein of interest. Notably, we also found that the auROC is not correlated with AlphaFold2's prediction confidence, as measured by the predicted local distance difference test (pLDDT; Fig EV3; Tunyasuvunakool *et al*, 2021). We found similar results using precision‐recall (PR) curves, which account for potential class imbalance by plotting the true‐positive rate against the positive predicted value (Figs EV4 and EV5). Here, the area under the PR curve (auPRC) can be interpreted as the ability of the model to correctly identify a true protein‐ligand binding interaction hit, and a horizontal line corresponding to the baseline fraction of hits indicates the performance expected from a random model. We found that the auPRC values across all 12 essential proteins ranged from 0.01 (*murC*) to 0.63 (*gyrAB*), with an average value of 0.21 which is marginally larger than the average baseline fraction of hits (0.16; Fig EV5). Similar to the auROC, the auPRC is not correlated with AlphaFold2's pLLDT (Fig EV3). 95% confidence intervals for the auROC and auPRC, as generated by bootstrapping, suggested that these values were robust to variability in the data (Table EV1). Hence, assessing model performance using the auROC and auPRC both indicated weak performance; we therefore aimed to further investigate the causes of the weak performance and methods of improving it.

### Comparing models based on AlphaFold2 structures with experimentally determined protein‐ligand complexes

Having shown that our molecular docking simulations with AlphaFold2‐predicted structures produce a mean auROC of 0.48 (mean auPRC of 0.21), we asked whether the weak performance was associated with the quality of the protein structures used. To address this, we repeated our docking simulations by docking each of the 218 active compounds to each of eight experimentally determined protein structures. These structures correspond to protein‐ligand complexes or single proteins deposited in the Protein Data Bank (PDB) and comprise *gyrA* (4CKL), *gyrB* (1AJ6), *dnaB* (6QEM), *ligA* (5TT5), *glmU* (1FWY), *murA* (1A2N), *murC* (1P3C), *murD* (2VTE), and *murF* (1GG4). Benchmarking model performance as before, we found that auROC values were quantitatively similar to before and ranged from 0.25 (*glmU*) to 0.69 (*gyrAB*), with a mean value of 0.46 (Fig 4D). Analogous results were found for auPRC values, which ranged from 0.03 (*ligA*) to 0.56 (*gyrAB*), with a mean value of 0.22 (Fig EV5). These findings show that molecular docking using AlphaFold2‐predicted structures is similar to using experimentally determined structures. This is consistent with previous assessments of AlphaFold's fidelity to experimentally determined protein structures (Jumper *et al*, 2021) and suggests that the weak performance of our model arises from the docking method, and not the quality of protein structures.

### Benchmarking and improving model performance using machine learning

Based on the weak performance of our molecular docking approach, we investigated ways in which performance could be improved. The foregoing platform uses AutoDock Vina, which employs empirical free energy scoring functions to evaluate docking poses. To investigate the effects of different docking methods on our benchmarking results, we extended our approach to utilize DOCK6.9 (Allen *et al*, 2015), a benchmarked open‐source program that uses force‐based scoring functions for docking. Furthermore, we augmented our approach with four different machine learning‐based scoring functions, RF‐Score (Ballester & Mitchell, 2010), RF‐Score‐VS (Wójcikowski *et al*, 2017), PLEC score (Wójcikowski *et al*, 2019), and NNScore (Durrant & McCammon, 2010). The RF‐Score and RF‐Score‐VS—a virtual screening adaptation of RF‐Score (Wójcikowski *et al*, 2017)—utilize random forests, or ensembles of decision trees, to predict protein‐ligand binding affinities. In contrast, the PLEC score employs extended connectivity fingerprints between protein‐ligand pairs, and the NNScore is based on an ensemble of neural networks. Recent studies have demonstrated improvements in prediction accuracy using the RF‐Score, RF‐Score‐VS, or NNScore to rescore docking poses predicted by AutoDock Vina (Li *et al*, 2015; Pereira *et al*, 2016; Wójcikowski *et al*, 2017), and prior work has shown that the PLEC score accurately estimates binding affinities in the PDBbind database (Wang *et al*, 2004; Wójcikowski *et al*, 2019) of empirical protein‐ligand interactions. Here, we employed each machine learning‐based scoring function, trained using the PDBbind v2016 or directory of useful decoys, enhanced (DUD‐E; Mysinger *et al*, 2012) databases, to rescore the docking poses predicted by AutoDock Vina. Of note, our test set shared only one overlapping protein‐ligand interaction, rifampicin bound to RNA polymerase (4KMU), with PDBbind v2016, and none with DUD‐E. Accordingly, testing these models on our enzymatic inhibition data accurately reflects what each model has learned.

**Figure 5:**
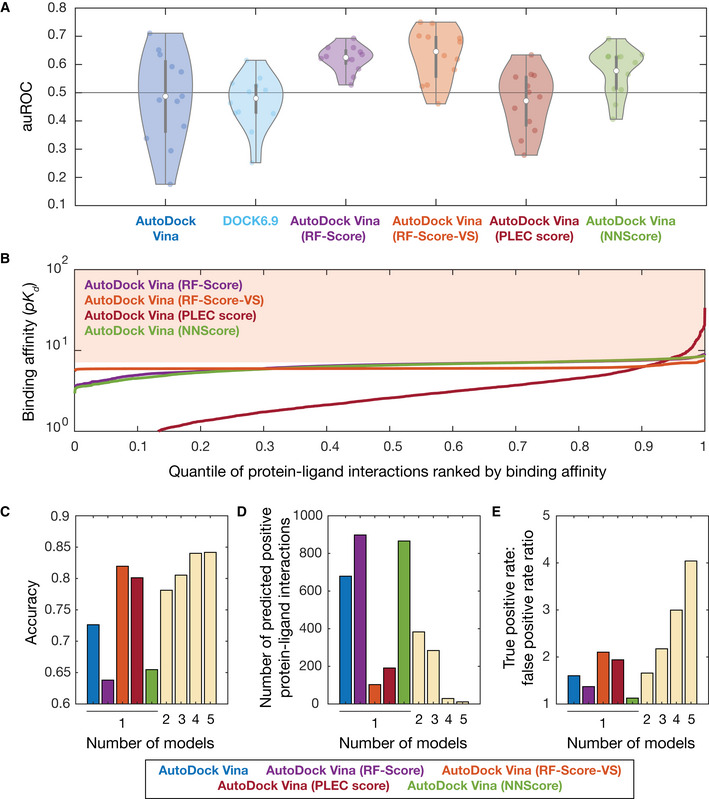
Benchmarking and improving model performance using machine learning AArea under the ROC curve (auROC) values for all 12 empirically tested essential proteins, across different molecular docking programs (AutoDock Vina and DOCK6.9) and different machine learning‐based pose scoring functions (RF‐Score, RF‐Score‐VS, PLEC score, and NNScore). White points indicate mean values, and gray bars indicate ranges of 25^th^ to 75^th^ percentile values (*Q*_1_ and *Q*_3_, respectively). The whiskers of the gray box plots indicate ranges of values not considered outliers, that is, those between *Q*_1_ – 1.5 × IQR and *Q*_3_ + 1.5 × IQR, where IQR = *Q*_3_ − *Q*_1_ is the interquartile range. The horizontal line at 0.5 indicates the benchmark generated by random guessing.BRank‐ordered binding affinities (*pK*_*d*_) for the protein‐ligand pairs modeled by applying machine learning‐based rescoring functions on AutoDock Vina poses. Curves are colored according to the rescoring function used in (A). The shaded area indicates a binding affinity threshold of > 7.C–EDependence of prediction accuracy, number of predicted positives (protein‐ligand interactions), and true‐positive rate/false‐positive rate on the number of models used. Single models, based on AutoDock Vina poses, are colored according to (A) as shown at bottom. Model predictions based on the following rescoring functions were ensembled in sequence: RF‐Score, NNScore, PLEC score, and RF‐Score‐VS.

Using DOCK6.9 and each machine learning‐based scoring function applied to AutoDock Vina poses, we predicted the binding affinity between each antibacterial compound and each of the 12 empirically tested essential proteins (Dataset EV5). We then benchmarked the performance of each approach as before and found average auROC values between 0.46 and 0.63 (Figs 5A and EV4). Docking with DOCK6.9 and rescoring AutoDock Vina poses with the PLEC score resulted, on average, in lower auROC values than those from AutoDock Vina alone, with auROC values of 0.46 (range of 0.25 to 0.61) for DOCK6.9 and 0.47 (range of 0.28 to 0.63) for the PLEC score (Figs 5A and EV4). In contrast, rescoring AutoDock Vina poses with the RF‐Score, RF‐Score‐VS, or NNScore led to improvements in model performance, with average auROC values of 0.62 (range of 0.53 to 0.69), 0.63 (range of 0.46 to 0.75), and 0.58 (range of 0.41 to 0.69), respectively (Figs 5A and EV4). Our results were similar for the auPRC, which exhibited a mean value as high as 0.24 when rescoring with the RF‐Score (Fig EV5), and are robust, as suggested by calculations of 95% confidence intervals for the auROC and auPRC values (Table EV1). These assessments of model performance indicate that certain machine learning‐based scoring functions improve prediction accuracy.

### Wisdom of crowds improves prediction accuracy and enriches for true positives

Building on our finding that certain machine learning‐based scoring functions increase the auROC and auPRC, we asked whether combining rescored models—a “wisdom of crowds” approach—could improve prediction accuracy and enrich for true positives given stringent binding affinity thresholds. We considered a stringent binding affinity threshold for the binding energies produced by AutoDock Vina (−7 kcal/mol), as before. For simplicity, we also considered a constant, stringent binding affinity threshold for the binding affinities produced by rescoring (*pK*_*d*_ > 7), which corresponds to the top 34, 3.9, 7.3, and 33% of the binding affinities predicted by rescoring AutoDock Vina poses with the RF‐Score, RF‐Score‐VS, PLEC score, and NNScore, respectively (Fig 5B). We ensembled our baseline AutoDock Vina predictions with those of the four machine learning‐based scoring functions used above by defining predicted protein‐ligand interactions as those satisfying the binding affinity thresholds across all models. Using this consensus approach, we found that prediction accuracy improved with the number of models used (Fig 5C), as may be expected from the corresponding decrease in the numbers of predicted protein‐ligand interactions (Fig 5D). Less expected is the fact that the ratio of the true‐positive rate to the false‐positive rate increased with the number of models used, from 1.60 with AutoDock Vina to 4.04 with all models applied (Fig 5E). This result is consistent with our finding of improved predictive power using certain machine learning‐based scoring functions. It further demonstrates that ensembling molecular docking with machine learning‐based models could allow one to better harness AlphaFold2‐predicted protein structures for drug screens.

## Discussion

The advent of AlphaFold2 and other deep neural networks for protein folding, such as RoseTTAFold (Baek *et al*, 2021; Humphreys *et al*, 2021), has been widely anticipated and celebrated in structural biology. An important use case of protein structure predictions is drug discovery, for which the availability of predicted protein structures for entire proteomes could enable the identification of molecular drug targets and drug mechanisms of action. Here, we benchmarked the use of AlphaFold2‐enabled molecular docking simulations to predict protein‐ligand interactions for antibiotic drug discovery. We combined protein structure predictions from AlphaFold2 with docking to predict protein‐ligand interactions between active and inactive antibacterial compounds found in a growth inhibition screen and 296 essential proteins in *E. coli* (Fig 1 and Box 1). We found that this approach predicts widespread promiscuity between both active and inactive compounds and essential protein targets, as well as known antibiotic‐protein interactions with weak‐to‐moderate true‐positive rates depending on the stringency of the binding affinity threshold chosen (Fig 2 and Datasets EV2 and EV3). We further assessed model performance by measuring *in vitro* enzymatic activity for 12 essential *E. coli* proteins (Fig 3). Although these measurements supported extensive promiscuity, they also demonstrated that this approach has weak predictive power in identifying true protein‐ligand interactions. True‐positive rates were comparable to false‐positive rates and were, on average, higher only for stringent binding affinity thresholds (Fig 4). Furthermore, assessing performance independently of binding affinity threshold using the auROC and auPRC, we found that this approach exhibits weak performance depending on the protein of interest and performs, on average, comparably to random. Consistent with previous assessments of AlphaFold2's fidelity to experimentally determined protein structures (Jumper *et al*, 2021), the limitations in performance revealed by our benchmarking did not arise from the AlphaFold2‐predicted protein structures themselves, as repeating our benchmarking with experimentally determined structures yielded similar performance (Fig 4). These findings suggest that, although AlphaFold2 can provide rich structural information, methods to more accurately model protein‐ligand interactions are needed to better harness AlphaFold2 for drug discovery.

Building on these findings and previous machine learning‐based approaches to modeling protein‐ligand interactions, we have shown that rescoring our docking poses with three machine learning‐based scoring functions improved prediction accuracy (Fig 5). In contrast, docking with a different platform, DOCK6.9, did not (Fig 5). While other software has been used for molecular docking in addition to AutoDock Vina and DOCK6.9, prior benchmarking studies using software including AutoDock Vina, DOCK6, Schrödinger's Glide, Surflex, and internal coordinate mechanics (ICM) have shown that performance is similar on the directory of useful decoys (DUD) dataset (Durrant *et al*, 2013; Pereira *et al*, 2016), suggesting that our findings do not depend on the docking software used. Thus, our work underscores that certain machine learning‐based approaches may better leverage structural information to predict protein‐ligand interactions. Consistent with this finding, refining our model predictions using consensus models and a wisdom‐of‐crowds approach increases the prediction accuracy and the ratio of true‐positive rate to false‐positive rate (Fig 5). These results should inspire the development of additional machine learning‐based approaches to complement the use of AlphaFold2 for drug discovery.

Although our original model with AutoDock Vina performs comparably to random on average, we note that its performance can vary significantly across the 12 essential proteins tested. For instance, our AutoDock Vina predictions for *gyrAB* exhibited true‐ and false‐positive rates of 0.79 and 0.62, respectively, for a binding affinity threshold of −7 kcal/mol, and an auROC of 0.71 (Fig 4). In contrast, the true‐ and false‐positive rates for *murC* were 0.20 and 0.61, respectively, given the same binding affinity threshold, and the auROC was 0.18 (Fig 4). We observed similar heterogeneity across the 12 essential proteins tested in rescored models (Figs 5 and EV4). In view of these observations, it is important to note that docking has been widely used in “one target, many compounds” approaches to enrich for screening hits (Lyu *et al*, 2019; Bender *et al*, 2021). Although docking and rescoring are necessarily imperfect, our benchmarking suggests that this approach may have acceptable predictive power for certain proteins, such as *gyrAB*, and could lead to improvements in hit rates in a large‐scale compound screen for inhibitors of these proteins. Indeed, combining AlphaFold2 with molecular docking and rescoring might aid in identifying binding compounds, enabling the discovery of next‐generation antibiotics in a protein‐dependent way. In such approaches, the quantity of interest is no longer the prediction accuracy across all the compounds tested, but rather the early enrichment for true positives (Bender *et al*, 2021). Our results suggest that, of relevance to both the reverse docking and the “one target, many compounds” cases, focusing on proteins for which performance is encouraging may better enable predictive, AlphaFold2‐driven approaches to drug discovery.

While molecular docking has developed and improved over the past 40 years (Kuntz *et al*, 1982; Meng *et al*, 2011), our study also indicates that further improvements in the modeling of protein‐ligand interactions are needed to better leverage AlphaFold2 for drug‐target identification. These efforts may include innovations in both protein structure prediction and docking methods. A clear limitation to AlphaFold2 is that it is unable to distinguish between the active and inactive conformations of a protein (Mullard, 2021). Concomitantly, limitations to the development of more accurate docking methods are the use of rigid protein docking in this and other benchmarking studies (Durrant *et al*, 2013; Pereira *et al*, 2016) and the scarcity of benchmarking datasets. Long molecular dynamics simulations that focus on a specific protein of interest could account for protein conformational changes that, in certain cases like AcrB, might be important for ligand binding (Vargiu & Nikaido, 2012). A standard benchmark dataset for evaluating docking performance has been DUD‐E (Mysinger *et al*, 2012); yet, this dataset has been evidenced to exhibit hidden bias, which may contribute to misleading machine learning models (Chen *et al*, 2019). More recent work, including the present study, has aimed to acquire and use empirical data to systematically test docking predictions, including datasets generated from the chemical synthesis of hundreds of compounds corresponding to both favorable and unfavorable binding affinity values (Lyu *et al*, 2019). Here, we have empirically measured the enzymatic activity of 12 essential *E. coli* proteins treated with each of 218 antibacterial compounds. These measurements include protein‐ligand pairs that were predicted to either interact or not interact by our approach. Additionally, we have assembled a dataset comprising 142 experimentally evidenced or inferred antibiotic binding interactions (Dataset EV3). These data can be used as additional resources to assess docking predictions, especially as applied to antibiotics and antibiotic mechanisms of action. Although measuring protein‐ligand binding interactions remains experimentally intensive—relying on methods including enzymatic activity assays, differential scanning fluorimetry, and surface plasmon resonance—the creation of datasets that correspond to real use cases of docking will contribute to the development and accurate benchmarking of more predictive docking approaches. This is especially timely, as benchmarking is emerging as a critical foundation of advances in machine learning.

Moving forward, we expect future work to refine our approach to further leverage advances in applying machine learning to molecular docking (Gentile *et al*, 2020; Stärk *et al*, 2022) and protein structure prediction in order to improve the prediction of protein‐ligand interactions for antibiotic drug discovery. We anticipate that similar approaches may be applied to identifying protein‐ligand interactions for diverse, urgently needed classes of drugs, including antineoplastic and antiviral compounds, and to the discovery and design of these drug classes. It would also be intriguing to characterize the proteins with structures that are uniquely provided by neural network predictions, and for which the prediction accuracy of docking can be shown to be encouraging. Large‐scale docking of compound libraries with such proteins might lead to improved hit rates in chemical screens, resulting in leads that target previously difficult‐to‐drug proteins. As our study demonstrates, harnessing AlphaFold2 for drug‐target prediction remains a nascent method, and realizing its potential for drug discovery will require substantive improvements in modeling protein‐ligand interactions. By benchmarking the performance of molecular docking simulations and demonstrating that machine learning‐based approaches can improve prediction accuracy, we anticipate that our study will inform the use of AlphaFold2 in drug discovery.

## Materials and Methods

### Bacterial strains and growth

*Escherichia coli* K‐12 BW25113 was used for all experiments described in this work. Cells were grown in liquid LB medium (product 244620, Becton Dickinson, Franklin Lakes, NJ). LB media containing 1.5% agar (Becton Dickinson 244520) was used to grow individual colonies.

### Growth inhibition screening of 39,128 chemical compounds

Compounds were sourced as two differently formatted libraries, one comprised of 96‐well plates and one comprised of 384‐well plates, in dimethyl sulfoxide (DMSO) at 5 mM concentration. The 96‐well library is an FDA‐approved drug library from MicroSource Discovery Systems (New Milford, CT) that was described in previous work (Stokes *et al*, 2020). The 384‐well library is an in‐house library of structurally diverse compounds with molecular weights between 40 and 4,200 Da. The libraries were kept in a −20°C freezer for long‐term storage. Similar to previous work determining growth inhibition (Stokes *et al*, 2020; Wong *et al*, 2021a, 2021b), *E. coli* BW25113 was grown overnight in liquid LB medium in 14 ml Falcon tubes at 37°C with shaking at 300 rpm in a light‐protected incubator, then diluted 1:10,000 in fresh liquid LB, and plated into clear 96‐well flat‐bottom plates (product 9018, Corning, Corning, NY) using 100 μl final working volumes or into clear 384‐well flat‐bottom plates (Corning 3680) using 50 μl final working volumes, with plate type chosen to match the format of the library screened. Compounds were added to a final concentration of 50 μM, and plates were incubated in sealed plastic bags at 37°C without shaking overnight (16 to 24 h). After incubation, the optical density (OD_600_) was read using a SpectraMax M3 plate reader (Molecular Devices, San Jose, CA) to quantify bacterial growth. Plate data were normalized by the interquartile mean of each plate to determine relative growth. All screens were performed in biological replicate, and the Pearson's correlation coefficient between relative growth values in replicates is *R* = 0.84 (*P* < 10^−14^), demonstrating good reproducibility between replicates. Chemical library information and all relative growth values are provided in Dataset EV1.

### Determination of essential genes in *Escherichia coli*

Essential genes in *E. coli* were compiled from previous studies based on transposon‐directed insertion site sequencing (Goodall *et al*, 2018) and CRISPR interference screening (Rousset *et al*, 2018, 2021). We shortlisted genes identified in at least two of these three studies, resulting in a total of 295 genes out of ∼ 4,000 total genes in *E. coli* (Blattner *et al*, 1997). In order to accommodate our comparisons to experimental data generated from the enzymatic inhibition of RNA polymerase, this list was supplemented with an additional gene, *rpoZ*, which was indicated to be essential in only one study (Goodall *et al*, 2018). A list of all 296 genes thus determined is provided in Dataset EV2. These genes were manually mapped to corresponding UniProt identifiers based on the *E. coli* K‐12 reference proteome (UniProt: UP000000625) and used to obtain the corresponding AlphaFold2‐predicted protein structures.

### Preparation of files for molecular docking

We used AutoDock Vina 1.2.0 (Eberhardt *et al*, 2021) to dock each of our 218 antibacterial compounds with each of the 296 essential *E. coli* proteins. Our approach is illustrated in Fig EV1A. Briefly, each of the 296 (unbound) protein structures was downloaded as a PDB file from the AlphaFold2 database publicly available at https://alphafold.ebi.ac.uk/download (Jumper *et al*, 2021; Varadi *et al*, 2022). Compounds were provided as simplified molecular‐input line‐entry system (SMILES) strings. As three‐dimensional structures are needed for docking, we used OpenBabel to convert the SMILES string of each compound into three‐dimensional chemical structures (represented in SDF format). The PDB (protein) and SDF (compound) files were taken as inputs to our docking approach.

We next used AutoDock Tools (Zhang *et al*, 2019) to prepare each protein and compound for docking, by converting each file into AutoDock Vina's PDBQT format. For compound preparation, hydrogen atoms were added at pH 7.4, and docking with water molecules was specified using the ‐w flag. Each compound was prepared using the following command on the compound's SDF file (“input.sdf”):

*mk_prepare_ligand.py ‐I input.sdf ‐o output.pdbqt –add_hydrogen –pH 7.4 ‐w*.

As indicated in Dataset EV2, docking with water molecules failed for 56 of the 218 active compounds (associated with an error of “water molecules could not be placed by AutoDock Vina”). Hence, for these 56 active compounds, no water molecules were explicitly added (the ‐w flag was removed from the command above), and the docking was repeated. We also note that one active compound contained a boron atom, which AutoDock Vina does not support (no force fields available). To enable docking of this compound, we replaced its boron atom with a carbon atom, as is often done in molecular docking (Tiwari *et al*, 2009). Each protein was prepared using the following command on the protein's PDB file (“input.pdb”):

*prepare_receptor ‐r input.pdb ‐o output.pdbqt*.

Following compound and protein preparation, active site coordinates must be specified for docking. Unless otherwise stated, we used blind docking, in which AutoDock Tools computes a prospective active site for each protein‐ligand pair. To perform blind docking, we first generated an affinity map for each protein‐ligand pair using the following command on the input PDBQT files (“compound.pdbqt” and “protein.pdbqt”):

*prepare_gpf4.py ‐l compound.pdbqt ‐r receptor.pdbqt ‐y ‐p ligand_types”A*,*NA*,*C*,*HD*,*N*,*O″ ‐o output.gpf*.

Then, AutoGrid 4 was used to determine the grid coordinates corresponding to each affinity map (“input.gpf”) as follows:

*autogrid4 ‐p input.gpf ‐l output.glg*.

The grid coordinates stored in each output GLG file, along with the corresponding compound and ligand PDBQT files, were used for docking.

### Molecular docking with AutoDock Vina

Docking was performed with a default exhaustiveness of 32, which specifies the number of runs that start with a random ligand conformation, and a default n_poses of 20, which specifies the final number of ligand poses to report. As positive controls for our docking simulations, we re‐docked 11 experimentally evidenced protein‐ligand complexes from the RCSB Protein Data Bank (Dataset EV2). For each complex, a PDB file containing both the protein and the ligand was downloaded from the Protein Data Bank. The PDB file was split into separate protein and ligand files using PyMol (version 2.0, Schrödinger Inc., New York, NY), then prepared, and docked as detailed above to predict binding poses. The predicted binding pose of each complex was superimposed with the experimentally determined structure using PyMol. We visually inspected the binding poses and found excellent agreement with the experimentally determined structures for all complexes, confirming the soundness of our docking approach for general screens. All binding affinities predicted by our docking simulations are reported in Dataset EV2.

To further improve the quality of our docking predictions, we searched the Protein Data Bank for protein‐ligand complexes that include the 12 empirically tested essential proteins. We found 6 complexes (Dataset EV2). We re‐docked the 6 protein‐ligand complexes as described above, validating good agreement between the predicted and experimentally determined binding poses. For each of these 6 protein structures, we repeated our docking simulations with each of the 218 antibacterial compounds, setting the active site to twice the linear dimensions of that in the experimentally determined protein‐ligand complex. This resulted in auROC values greater than those obtained with blind docking for two proteins, MurA and GyrAB. Consequently, the reported binding affinities for MurA and GyrAB are those predicted by simulations using these empirically determined active sites.

### DNA gyrase inhibition assay

Inhibition of *E. coli* DNA gyrase (GyrA‐GyrB complex; DNA topoisomerase II) supercoiling was assessed using an *in vitro* assay developed by ProFoldin (Hudson, MA), following the manufacturer's instructions with some modifications. The assay is based on the principle that supercoiled DNA and relaxed DNA yield different fluorescent intensities when interacting with the fluorescent dye H19, with relaxed DNA suppressing the fluorescent intensity more than the supercoiled form in the presence of magnesium. Each reaction was performed using 20 μl of reaction mixture including 12 μl ultrapure Milli‐Q water, 2 μl of 10× buffer T2, 2 μl of 10× relaxed DNA, 2 μl of 10× enzyme, and 2 μl of 10 mM ATP, resulting in final concentrations of 20 mM Tris–HCl (pH 8.0), 35 mM NH_4_OAc, 4.6% glycerol, 1 mM dithiothreitol, 0.005% Brij‐35, 8 mM MgCl_2_, 25 μg/ml relaxed plasmid DNA, 1 mM ATP, and 20 nM DNA gyrase. Eighteen μl of diluted buffer containing enzyme and ATP was plated into standard black 384‐well plates (Corning 3575). Where applicable, 0.4 μl of test compound (or DMSO as a negative control) was added, and plates were incubated at room temperature for at least 5 min. Two μl of 10× relaxed DNA was then added to each reaction. For generating standard curves, the amount of substrate (relaxed DNA) added was decreased in proportion to activity. Plates were incubated at 37°C for 2 h. The provided 10× H19 dilution buffer was diluted 10‐fold with ultrapure Milli‐Q water, and the provided H19 dye was diluted 1,500× with 1× H19 dilution buffer. After incubation, 80 μl of diluted H19 dye was added to each reaction, and mixtures were incubated at room temperature for 5 min. The fluorescence excitation/emission at 485/535 nm was then measured using a SpectraMax M3 plate reader. For each sample, activity was determined by linear interpolation with respect to the standard curves provided that the resulting fluorescence intensity value fell within the standard curve range. Otherwise, fluorescence intensity values below that of the zero standard were clipped to that of the zero standard, and fluorescence intensity values above that of the highest standard were linearly extrapolated with respect to that of the highest standard.

### DNA primase inhibition assay

Inhibition of *E. coli* DNA primase (DnaG)—which synthesizes RNA primers at the DNA replication fork where DnaB unwinds the double‐stranded DNA—was assessed using an *in vitro* assay developed by ProFoldin (Hudson, MA), following the manufacturer's instructions with some modifications. The assay is based on the measurement of the RNA primers synthesized by DNA primase in the presence of DNA template and NTPs. For screening experiments, reactions were performed using 40 μl of reaction mixture including 24 μl ultrapure Milli‐Q water, 4 μl of 10× assay buffer, 4 μl of 10× DNA template, 4 μl of 10× enzyme, and 4 μl of 10× NTP mix, resulting in final concentrations of 10 mM HEPES (pH 7.5), 5 mM magnesium sulfate, 0.5 mM dithiothreitol, 0.003% Brij‐35, 100 nM DNA, 0.5 mM NTPs, and 100 nM enzyme. Thirty‐six μl of diluted buffer containing enzyme and NTP mix was plated into standard black 384‐well plates (Corning 3575). Where applicable, 0.8 μl of test compound (or DMSO as a negative control) was added, and plates were incubated at room temperature for at least 5 min. Four μl of 10× DNA template was then added to each reaction. For generating standard curves, the amount of substrate (DNA template) added was decreased in proportion to activity. Plates were incubated at 37°C for 2 h. The provided 10× fluorescence dye was diluted 10‐fold with ultrapure Milli‐Q water. After incubation, 60 μl of 1× dye was added to each reaction, and mixtures were incubated at room temperature for 5 min. The fluorescence excitation/emission at 485/535 nm was then measured using a SpectraMax M3 plate reader. For each sample, activity was determined by linear interpolation with respect to the standard curves provided that the resulting fluorescence intensity value fell within the standard curve range. Otherwise, fluorescence intensity values below that of the zero standard were clipped to that of the zero standard, and fluorescence intensity values above that of the highest standard were linearly extrapolated with respect to that of the highest standard. For subsequent validation dose–response experiments, half the indicated reaction volumes—that is, 20 μl for each reaction mixture—was used, and 40 μl of 1× dye was added to each reaction.

### DNA helicase inhibition assay

Inhibition of *E. coli* DnaB (DnaB)—which hydrolyzes ATP to carry out the DNA unwinding required by the DNA replication process—was assessed using an *in vitro* assay developed by ProFoldin (Hudson, MA), following the manufacturer's instructions. The assay is based on the measurement of inhibition of the ATPase activity of DNA helicase, particularly the detection of the phosphate produced by the ATP hydrolysis reaction in the presence of DNA. For each 10 assay reactions, 297 μl of premix comprising 261 μl of ultrapure Milli‐Q water, 33 μl of 10× assay buffer, and 3.3 μl of 100× DNA helicase were prepared. Additionally, 33 μl of 10× enzyme substrate comprising 3.3 μl of 100× ATP, 3.3 μl of 100× DNA, and 26.4 μl of ultrapure Milli‐Q water were prepared. The final concentrations of reagents in each assay are as follows: 20 mM HEPES (pH 7.5), 20 mM potassium glutamate, 1 mM dithiothreitol, 0.005% Triton X‐100, 10 mM MgCl_2_, 250 nM DNA, 0.25 mM ATP, and 200 nM DNA helicase. For each reaction, 26.4 μl of the premix was plated into standard clear 384‐well plates (ThermoFisher 242757). Where applicable, 0.6 μl of test compound (or DMSO as a negative control) was added, and plates were incubated at room temperature for at least 5 min. Three μl of 10× enzyme substrate was then added to each reaction. For generating standard curves, the amount of 10× enzyme substrate added was decreased in proportion to activity. Plates were incubated at 37°C for 2 h. After incubation, 45 μl of the provided MPA3000 dye was added to each reaction, and mixtures were incubated at room temperature for 5 min. The absorbance at 650 nm was then measured using a SpectraMax M3 plate reader. For each sample, activity was determined by linear interpolation with respect to the standard curves provided that the resulting absorbance value fell within the standard curve range. Otherwise, absorbance values below that of the zero standard were clipped to that of the zero standard, and absorbance values above that of the highest standard were linearly extrapolated with respect to that of the highest standard.

### NAD^+^‐dependent DNA ligase inhibition assay

Inhibition of *E. coli* NAD^+^‐dependent DNA ligase (LigA)—which catalyzes the formation of phosphodiester linkages between 5′‐phosphoryl and 3′‐hydroxyl groups in double‐stranded DNA using NAD^+^ as a coenzyme and as the energy source for the reaction—was assessed using an *in vitro* assay developed by ProFoldin (Hudson, MA), following the manufacturer's instructions with some modifications. The assay is based on the measurement of the DNA ligase product in which the diphosphodiester bond is formed at the single‐strand break of a duplex DNA substrate. Reactions were performed using 20 μl of reaction mixture including 13.8 μl ultrapure Milli‐Q water, 2 μl of 10× buffer LS, 2 μl of 10× DNA, 0.2 μl of 100× enzyme, and 2 μl of 1 mM NAD^+^. Twenty μl of diluted buffer containing enzyme and NAD^+^ was plated into standard black 384‐well plates (Corning 3575). Where applicable, 0.4 μl of test compound (or DMSO as a negative control) was added, and plates were incubated at room temperature for at least 5 min. Two μl of 10× DNA was then added to each reaction. For generating standard curves, the amount of substrate (DNA) added was decreased in proportion to activity. Plates were incubated at 37°C for 2 h. After incubation, 70 μl of Reagent T, then 10 μl of the provided fluorescent dye (diluted to 1× in ultrapure Milli‐Q water) was added to each reaction, and mixtures were incubated at room temperature for 15 min. The fluorescence excitation/emission at 485/535 nm was then measured using a SpectraMax M3 plate reader. For each sample, activity was determined by linear interpolation with respect to the standard curves provided that the resulting fluorescence intensity value fell within the standard curve range. Otherwise, fluorescence intensity values below that of the zero standard were clipped to that of the zero standard, and fluorescence intensity values above that of the highest standard were linearly extrapolated with respect to that of the highest standard.

### DNA polymerase III inhibition assay

Inhibition of *E. coli* DNA polymerase III's catalytic α subunit (DnaE)—which synthesizes DNA using the RNA primer made by the DNA primase at the DNA replication fork—was assessed using an *in vitro* assay developed by ProFoldin (Hudson, MA), following the manufacturer's instructions with some modifications. Reactions were performed using 20 μl of reaction mixture including 12 μl ultrapure Milli‐Q water, 2 μl of 10× buffer DP, 2 μl of 10× DNA, 2 μl of 10× enzyme, and 2 μl of 10× dNTP mix. Twenty μl of diluted buffer containing enzyme and dNTP mix was plated into standard black 384‐well plates (Corning 3575). Where applicable, 0.4 μl of test compound (or DMSO as a negative control) was added, and plates were incubated at room temperature for at least 5 min. Two μl of 10× DNA was then added to each reaction. For generating standard curves, the amount of substrate (DNA) added was decreased in proportion to activity. Plates were incubated at 37°C for 2 h. After incubation, 40 μl of the provided fluorescent dye (diluted to 1× in ultrapure Milli‐Q water) was added to each reaction, and mixtures were incubated at room temperature for 5 min. The fluorescence excitation/emission at 485/535 nm was then measured using a SpectraMax M3 plate reader. For each sample, activity was determined by linear interpolation with respect to the standard curves provided that the resulting fluorescence intensity value fell within the standard curve range. Otherwise, fluorescence intensity values below that of the zero standard were clipped to that of the zero standard, and fluorescence intensity values above that of the highest standard were linearly extrapolated with respect to that of the highest standard.

### RNA polymerase inhibition assay

Inhibition of *E. coli* RNA polymerase (RpoA, RpoB, RpoC, RpoZ, RpoE holoenzyme, with a molecular mass of ∼ 390 kDa)—which synthesizes mRNA, tRNA, and rRNA in cells—was assessed using an *in vitro* assay developed by ProFoldin (Hudson, MA), following the manufacturer's instructions. The assay is based on the measurement of the RNA synthesized by the RNA polymerase using a DNA template. Each reaction was performed using 30 μl of reaction mixture including 18 μl ultrapure Milli‐Q water, 3 μl of 10× buffer, 3 μl of 10× DNA template, 3 μl of 10× enzyme, and 3 μl of 10× NTP mix. Twenty‐seven μl of diluted buffer containing enzyme and NTP mix was plated into standard black 384‐well plates (Corning 3575). Where applicable, 0.6 μl of test compound (or DMSO as a negative control) was added, and plates were incubated at room temperature for at least 5 min. Three μl of 10× DNA template was then added to each reaction. For generating standard curves, the amount of substrate (DNA template) added was decreased in proportion to activity. Plates were incubated at 37°C for 2 h. After incubation, 30 μl of the provided fluorescent dye was added to each reaction, and mixtures were incubated at room temperature for 5 min. The fluorescence excitation/emission at 485/535 nm was then measured using a SpectraMax M3 plate reader. For each sample, activity was determined by linear interpolation with respect to the standard curves provided that the resulting fluorescence intensity value fell within the standard curve range. Otherwise, fluorescence intensity values below that of the zero standard were clipped to that of the zero standard, and fluorescence intensity values above that of the highest standard were linearly extrapolated with respect to that of the highest standard.

### Guanylate kinase inhibition assay

Inhibition of *E. coli* guanylate kinase (*gmk*)—which catalyzes the ATP‐dependent phosphorylation of GMP into GDP in order to recycle GMP and cGMP—was assessed using an *in vitro* assay developed by ProFoldin (Hudson, MA), following the manufacturer's instructions. Reactions were performed using 30 μl of reaction mixture including 12 μl ultrapure Milli‐Q water, 3 μl of 10× reaction buffer, 3 μl of 10× GMP, 3 μl of 10× ATP, and 3 μl of 10× kinase. 24 μl of diluted buffer containing enzyme, GMP, and ATP was plated into standard clear 384‐well plates (ThermoFisher 242757). Where applicable, 0.6 μl of test compound (or DMSO as a negative control) was added, and plates were incubated at room temperature for at least 5 min. Three μl of 10× GMP was then added to each reaction, and the reaction was incubated at room temperature for 2 min. To each reaction, 3 μl of 10× MUK reagent A was added, followed by 3 μl of MUK reagent B. For generating standard curves, the amount of substrate (GMP) added was decreased in proportion to activity. Plates were incubated at 37°C for 2 h. After incubation, 30 μl of the provided fluorescent dye (diluted to 1× in ultrapure Milli‐Q water) was added to each reaction, and mixtures were incubated at room temperature for 5 min. The fluorescence excitation/emission at 485/535 nm was then measured using a SpectraMax M3 plate reader. For each sample, activity was determined by linear interpolation with respect to the standard curves provided that the resulting fluorescence intensity value fell within the standard curve range. Otherwise, fluorescence intensity values below that of the zero standard were clipped to that of the zero standard, and fluorescence intensity values above that of the highest standard were linearly extrapolated with respect to that of the highest standard.

### GlmU inhibition assay

Inhibition of *E. coli* GlmU (UDP‐N‐acetylglucosamine pyrophosphorylase)—which transfers acetyl and uridyl groups to glucosamine‐1‐P, generating UDP‐GlcNAc (a peptidoglycan precursor)—was assessed using an *in vitro* assay developed by ProFoldin (Hudson, MA), following the manufacturer's instructions. The assay is based on the measurement of the pyrophosphate generated from the GlmU reaction. For each 10 assay reactions, 297 μl of premix comprising 257.4 μl of ultrapure Milli‐Q water, 33 μl of 10× assay buffer, and 3.3 μl of 100× GlmU (500 nM) were prepared. Additionally, 33 μl of 10× enzyme substrate comprising 3.3 μl of the provided 100× enzyme substrate (2.5 mM glucosamine‐1‐P, 2.5 mM acetyl‐CoA, and 2.5 mM UTP) and 29.7 μl of ultrapure Milli‐Q water were prepared. For each reaction, 26.4 μl of the premix was plated into standard clear 384‐well plates (ThermoFisher 242757). Where applicable, 0.6 μl of test compound (or DMSO as a negative control) was added, and plates were incubated at room temperature for at least 5 min. Three μl of 10× enzyme substrate was then added to each reaction. For generating standard curves, the amount of 10× enzyme substrate added was decreased in proportion to activity. Plates were incubated at 37°C for 2 h. After incubation, 45 μl of the provided MPA3000 dye was added to each reaction, and mixtures were incubated at room temperature for 5 min. The absorbance at 650 nm was then measured using a SpectraMax M3 plate reader. For each sample, activity was determined by linear interpolation with respect to the standard curves provided that the resulting absorbance value fell within the standard curve range. Otherwise, absorbance values below that of the zero standard were clipped to that of the zero standard, and absorbance values above that of the highest standard were linearly extrapolated with respect to that of the highest standard.

### MurA inhibition assay

Inhibition of *E. coli* MurA (UDP‐N‐acetylglucosamine enolpyruvyl transferase)—which transfers enolpyruvate from phosphoenolpyruvate (PEP) to uridine diphospho‐N‐acetylglucosamine (UNAG), generating enolpyruvyl‐UDP‐N‐acetylglucosamine (EP‐UNAG) and inorganic phosphate—was assessed using an *in vitro* assay developed by ProFoldin (Hudson, MA), following the manufacturer's instructions. The assay is based on the measurement of the inorganic phosphate generated from the MurA reaction. For each 10 assay reactions, 297 μl of premix comprising 261 μl of ultrapure Milli‐Q water, 33 μl of 10× assay buffer, and 3.3 μl of 100× MurA (5 μM) were prepared. Additionally, 33 μl of 10× enzyme substrate comprising 3.3 μl of 100× PEP, 3.3 μl of 100× UDP‐N‐acetylglucosamine (UGN), and 26.4 μl of ultrapure Milli‐Q water were prepared. For each reaction, 26.4 μl of the premix was plated into standard clear 384‐well plates (ThermoFisher 242757). Where applicable, 0.6 μl of test compound (or DMSO as a negative control) was added, and plates were incubated at room temperature for at least 5 min. Three μl of 10× enzyme substrate was then added to each reaction. For generating standard curves, the amount of 10× enzyme substrate added was decreased in proportion to activity. Plates were incubated at 37°C for 2 h. After incubation, 45 μl of the provided MPA3000 dye was added to each reaction, and mixtures were incubated at room temperature for 5 min. The absorbance at 650 nm was then measured using a SpectraMax M3 plate reader. For each sample, activity was determined by linear interpolation with respect to the standard curves provided that the resulting absorbance value fell within the standard curve range. Otherwise, absorbance values below that of the zero standard were clipped to that of the zero standard, and absorbance values above that of the highest standard were linearly extrapolated with respect to that of the highest standard.

### MurC inhibition assay

Inhibition of *E. coli* MurC (UDP‐N‐acetylmuramic acid:L‐alanine ligase)—which catalyzes the addition of L‐alanine into the nucleotide precursor UDP‐MurNAc, generating UDP‐MurNAc‐L‐Ala and whose ligation reaction is coupled to the hydrolysis of ATP, forming ADP and inorganic phosphate—was assessed using an *in vitro* assay developed by ProFoldin (Hudson, MA), following the manufacturer's instructions. The assay is based on the measurement of the inorganic phosphate generated from the MurC reaction. For each 10 assay reactions, 297 μl of premix comprising 261 μl of ultrapure Milli‐Q water, 33 μl of 10× assay buffer, and 3.3 μl of 100× MurC (5 μM) were prepared. Additionally, 33 μl of 10× enzyme substrate comprising 3.3 μl of 100× UDP‐MurNAc (4 mM), 3.3 μl of 100× L‐Ala (4 mM), 3.3 μl of 100× ATP (10 mM), and 23.1 μl of ultrapure Milli‐Q water were prepared. For each reaction, 26.4 μl of the premix was plated into standard clear 384‐well plates (ThermoFisher 242757). Where applicable, 0.6 μl of test compound (or DMSO as a negative control) was added, and plates were incubated at room temperature for at least 5 min. Three μl of 10× enzyme substrate was then added to each reaction. For generating standard curves, the amount of 10× enzyme substrate added was decreased in proportion to activity. Plates were then incubated at 37°C for 2 h. After incubation, 45 μl of the provided MPA3000 dye was added to each reaction, and mixtures were incubated at room temperature for 5 min. The absorbance at 650 nm was then measured using a SpectraMax M3 plate reader. For each sample, activity was determined by linear interpolation with respect to the standard curves provided that the resulting absorbance value fell within the standard curve range. Otherwise, absorbance values below that of the zero standard were clipped to that of the zero standard, and absorbance values above that of the highest standard were linearly extrapolated with respect to that of the highest standard.

### MurD inhibition assay

Inhibition of *E. coli* MurD (UDP‐N‐acetylmuramoylalanine:D‐glutamate ligase)—which catalyzes the addition of D‐glutamic acid to UDP‐MurNAc‐L‐Ala, generating UDP‐MurNAc‐dipeptide, and whose ligation reaction is coupled to the hydrolysis of ATP, forming ADP and inorganic phosphate—was assessed using an *in vitro* assay developed by ProFoldin (Hudson, MA), following the manufacturer's instructions. The assay is based on the measurement of the inorganic phosphate generated from the MurD reaction. For each 10 assay reactions, 297 μl of premix comprising 261 μl of ultrapure Milli‐Q water, 33 μl of 10× assay buffer, and 3.3 μl of 100× MurD (2 μM) were prepared. Additionally, 33 μl of 10× enzyme substrate comprising 3.3 μl of 100× UDP‐MurNAc‐L‐Ala (UMA), 3.3 μl of 100× D‐Glu, 3.3 μl of 100× ATP, and 23.1 μl of ultrapure Milli‐Q water were prepared. For each reaction, 26.4 μl of the premix was plated into standard clear 384‐well plates (ThermoFisher 242757). Where applicable, 0.6 μl of test compound (or DMSO as a negative control) was added, and plates were incubated at room temperature for at least 5 min. Three μl of 10× enzyme substrate was then added to each reaction. For generating standard curves, the amount of 10× enzyme substrate added was decreased in proportion to activity. Plates were then incubated at 37°C for 2 h. After incubation, 45 μl of the provided MPA3000 dye was added to each reaction, and mixtures were incubated at room temperature for 5 min. The absorbance at 650 nm was then measured using a SpectraMax M3 plate reader. For each sample, activity was determined by linear interpolation with respect to the standard curves provided that the resulting absorbance value fell within the standard curve range. Otherwise, absorbance values below that of the zero standard were clipped to that of the zero standard, and absorbance values above that of the highest standard were linearly extrapolated with respect to that of the highest standard.

### MurF inhibition assay

Inhibition of *E. coli* MurF (UDP‐N‐acetylmuramoyl‐tripeptide:D‐alanyl‐D‐alanine ligase)—which catalyzes the addition of D‐Ala‐D‐Ala to UDP‐MurNAc‐tripeptide, and whose ligation reaction is coupled to the hydrolysis of ATP, forming ADP and inorganic phosphate—was assessed using an *in vitro* assay developed by ProFoldin (Hudson, MA), following the manufacturer's instructions. The assay is based on the measurement of the inorganic phosphate generated from the MurF reaction. For each 10 assay reactions, 297 μl of premix comprising 261 μl of ultrapure Milli‐Q water, 33 μl of 10× assay buffer, and 3.3 μl of 100× MurF (2 μM) were prepared. Additionally, 33 μl of 10× enzyme substrate comprising 3.3 μl of 100× UDP‐MurNAc‐tripeptide (UMAG‐DAP), 3.3 μl of 100× D‐Ala‐D‐Ala, 3.3 μl of 100× ATP, and 23.1 μl of ultrapure Milli‐Q water were prepared. For each reaction, 26.4 μl of the premix was plated into standard clear 384‐well plates (ThermoFisher 242757). Where applicable, 0.6 μl of test compound (or DMSO as a negative control) was added, and plates were incubated at room temperature for at least 5 min. Three μl of 10× enzyme substrate was then added to each reaction. For generating standard curves, the amount of 10× enzyme substrate added was decreased in proportion to activity. Plates were then incubated at 37°C for 2 h. After incubation, 45 μl of the provided MPA3000 dye was added to each reaction, and mixtures were incubated at room temperature for 5 min. The absorbance at 650 nm was then measured using a SpectraMax M3 plate reader. For each sample, activity was determined by linear interpolation with respect to the standard curves provided that the resulting absorbance value fell within the standard curve range. Otherwise, absorbance values below that of the zero standard were clipped to that of the zero standard, and absorbance values above that of the highest standard were linearly extrapolated with respect to that of the highest standard.

### Analysis of *in vitro* protein inhibition experiments

For proteins with subunits (GyrAB and RpoABCEZ), a binding affinity was assigned to the protein by taking the minimum binding affinity across all subunits. To estimate IC_50_ values in the dose–response curves shown in Fig EV2, we used nonlinear least‐squares fitting (the lsqcurvefit function in MATLAB, Mathworks, Natick, MA) to fit mean activity values to Hill functions of the form

$$H\left(x\right)=b_{0}+\frac{mx^{\beta }}{x_{0.5}^{\beta }+x^{\beta }},$$

while enforcing *H*(0) = 1 and *H* ≥ 0 for all *x*. IC_50_ values were determined by numerically solving the best‐fit Hill function for *x* given *H*(*x*) = 0.5.

### Antibiotics and antibacterial compounds

Compounds were sourced in bulk for additional validation in the dose–response experiments shown in Fig EV2. Ceftibuten hydrate (product 25334, Cayman Chemical Company, Ann Arbor, MI), ceftiofur sodium (product HY‐B0898, MedChemExpress, Princeton, NJ), demeclocycline hydrochloride (MedChemExpress HY‐17560), oxytetracycline (Cayman 18076), minocycline hydrochloride hydrate (Cayman 14454), rifampicin (Cayman 14423), rifapentine (MedChemExpress HY‐B0269), WIN 64338 hydrochloride (BRD‐K22662557; product 1057, Bio‐Techne, Minneapolis, MN), mitoquinone mesylate (BRD‐K00003362; product S8978, Selleck Chemicals, Houston, TX), and PF‐477736 (BRD‐K03063480; Selleck S2904) were dissolved and serially diluted in DMSO (MilliporeSigma D5879). Ciprofloxacin (MilliporeSigma 17850) was dissolved in dilute acid (0.1 M HCl) and serially diluted in ultrapure Milli‐Q water.

### t‐SNE visualization of protein‐ligand fingerprints

For each docked protein‐ligand pair, we used the Open Drug Discovery Toolkit (ODDT)'s SimpleInteractionFingerprint() function to generate an amino acid‐based fingerprint of constant size across all proteins and ligands (Wójcikowski *et al*, 2015). t‐SNE was performed using sklearn's TSNE() function with a perplexity parameter of 30 and the Jaccard metric as the distance.

### Docking using experimentally determined protein structures

To compare our AlphaFold‐based docking predictions to those from experimentally determined structures, we searched the Protein Data Bank for protein‐ligand complexes that include the 12 empirically tested essential proteins. We found 3 protein structures (*dnaB*, 6QEM; *murC*, 1P3C; *murF*, 1GG4) in addition to the 6 protein‐ligand complexes detailed above in *Molecular docking with AutoDock Vina*. We repeated our docking simulations using each protein structure as described above in *Molecular docking with AutoDock Vina*. Of note, blind docking was used for protein structures without ligands. For each protein‐ligand complex, the active site was set to twice the linear dimensions of that in the experimentally determined structure, as detailed above in *Molecular docking with AutoDock Vina*.

### Calculation of ROC curves, PR curves, and confidence intervals

The receiver operating characteristic (ROC) curves and area under the ROC curve (auROC) values shown in Figs 4 and 5, and EV4 were calculated using the perfcurve function in MATLAB. For each protein‐ligand pair, the ground truth value was obtained by binarizing the relative enzymatic activity value (1 if the relative enzymatic activity was less than 0.5 in both biological replicates, and 0 otherwise). The input scores, which represent classifier predictions, were taken to be −1 times the binding affinity estimates (for AutoDock Vina or DOCK6.9 predictions) or equal to the binding affinity estimates (for AutoDock Vina predictions rescored using RF‐Score, PLEC score, or NNScore). 95% confidence intervals for the auROC and auPRC, as shown in Table EV1, were calculated by bootstrapping with 100 samples for each protein. Each sample was of size equal to the number of tested compounds (218) and was sampled with replacement.

### Molecular docking simulations with DOCK6.9

As described in the main text, we employed another docking platform, DOCK6.9 (Allen *et al*, 2015), to further benchmark our docking predictions. Our approach is illustrated in Fig EV1B, and docking simulations using DOCK6.9 were performed only for the 12 empirically tested essential proteins in this work. First, compounds were converted into three‐dimensional structures (MOL2 format) using OpenBabel. Next, we prepared each protein and each ligand for docking. We used a Python script that employs the *DockPrep* function from UCSF Chimera (Pettersen *et al*, 2004; Allen, 2018) to add hydrogens and partial charges. We found that 65 antibacterial compounds contained unknown atom names and/or types (as indicated in Dataset EV5). For these compounds, hydrogens and partial charges were manually added using *DockPrep*, available under Tools → Structure Editing in Chimera's graphical user interface. Each protein and each compound structure were then saved in MOL2 format.

In DOCK6.9, the molecular surface of a protein contains information about the van der Waals forces experienced in any ligand interaction. The molecular surface and active site of each protein are needed for docking. In order to generate the molecular surface, we used the *WriteDMS* function from UCSF Chimera, which reports the protein regions accessible to ligand binding in a DMS file. Given the DMS file, a sphere generation function (*Sphgen*) was used to generate spheres within empty spaces and/or hydrophobic pockets of each protein. The generated spheres represent plausible locations for ligand binding, and this blind docking approach was employed for all 12 essential proteins of interest. Spheres were filtered by setting the minimum and maximum radii to 1.4 and 4.0 Å, respectively, and an output SPH file comprising all clusters within a protein was generated. The largest cluster, which typically contains the predicted active site, was retained, and all other clusters were manually removed from the SPH file.

As a final step before docking, it is convenient to precompute an energy grid centered on the sphere cluster using the *Grid* function; doing so reduces the number of computations required for docking. The resulting energy scoring function estimates molecular mechanics interaction energies, comprising van der Waals and electrostatic components, at a pre‐specified grid spacing of 4.0 Å. Finally, we used flex docking, in which the ligand has full rotational freedom, in DOCK6.9 to predict binding poses and binding affinities. The binding affinity of each protein‐ligand pair of interest is represented by DOCK6.9's grid score and reported in Dataset EV5. As previously shown to occur when using DOCK (Jiang & Rizzo, 2015; Zhang *et al*, 2020), the software may fail to dock protein‐ligand pairs due to the inability to complete growth in its anchor‐and‐grow search algorithm. Consistent with these reports, we found that DOCK failed to dock a subset (26%) of the protein‐ligand pairs of interest. These failures are reported to have a binding affinity of 0 kcal/mol in Dataset EV5 and were removed from our analysis for calculation of the auROC values shown in Fig 5. We note here that, as additional controls for our modeling platform using DOCK6.9, we re‐docked 7 protein‐ligand complexes from the Protein Data Bank as controls. As detailed in *Molecular docking with AutoDock Vina*, these protein‐ligand complexes include seven of the empirically tested essential proteins. The resulting binding pose of each protein‐ligand complex was confirmed to be in good agreement with the experimentally evidenced pose.

### Rescoring docking poses with machine learning‐based scoring functions

We rescored the docking poses generated by AutoDock Vina using the Open Drug Discovery Toolkit (ODDT)'s implementations (Wójcikowski *et al*, 2015) of RF‐Score (Ballester & Mitchell, 2010), PLEC score (Wójcikowski *et al*, 2019), and NNScore (Durrant & McCammon, 2010). The PDBQT files of all docked ligands and all 12 essential proteins tested were read using ODDT. For rescoring with the PLEC score, the underlying model used was linear regression, and the parameters used were depth_protein = 5, depth_ligand = 1, and size = 65,536. These scoring functions were trained with protein‐ligand interaction data from PDBbind v2016, using ODDT's internal scorer.train() function. RF‐Score‐VS (Wójcikowski *et al*, 2017) is not implemented in ODDT, but is available in binary format, trained on the DUD‐E dataset (Mysinger *et al*, 2012), from https://github.com/oddt/rfscorevs_binary. The binary file was executed using the PDBQT files of all docked ligands, and all 12 essential proteins tested as inputs. The binding affinity (*pK*_*d*_) predictions generated by all rescoring methods are reported in Dataset EV5.

### Calculation of pLDDT values

For all 12 empirically tested essential proteins, AlphaFold2‐predicted protein structures in PDB format were downloaded from https://alphafold.ebi.ac.uk. The mean predicted local distance difference test (pLDDT) value of each protein was extracted and calculated from the B‐factor column containing the pLDDT value per atom and per residue (Tunyasuvunakool *et al*, 2021). For each protein, the protein‐averaged pLDDT value shown in Fig EV3 was calculated as the average pLDDT value across all residues.

## Author contributions

**Felix Wong:** Conceptualization; data curation; software; formal analysis; supervision; validation; investigation; visualization; methodology; writing – original draft; writing – review and editing. **Aarti Krishnan:** Data curation; software; formal analysis; validation; investigation; visualization; methodology; writing – original draft; writing – review and editing. **Erica J Zheng:** Data curation; investigation; writing – original draft; writing – review and editing. **Hannes Stärk:** Formal analysis; writing – review and editing. **Abigail L Manson:** Software; formal analysis; writing – original draft; writing – review and editing. **Ashlee M Earl:** Software; formal analysis; writing – original draft; writing – review and editing. **Tommi Jaakkola:** Formal analysis; writing – review and editing. **James J Collins:** Conceptualization; supervision; funding acquisition; writing – original draft; writing – review and editing.

In addition to the CRediT author contributions listed above, the contributions in detail are:

FW and JJC conceived and supervised the research. FW and AK designed models and experiments, performed experiments and analysis, and wrote the paper. EJZ performed experiments. HS, ALM, AME, and TJ performed analysis and assisted with data interpretation. All authors assisted with manuscript editing.

## Disclosure and competing interests statement

JJC is scientific co‐founder and scientific advisory board chair of EnBiotix, an antibiotic drug discovery company, and PhareBio, a non‐profit venture focused on antibiotic drug development. The remaining authors declare no competing interests. JJC is an editorial advisory board member. This has no bearing on the editorial consideration of this article for publication.

## Supporting information



Expanded View Figures PDF

Table EV1

Dataset EV1

Dataset EV2

Dataset EV3

Dataset EV4

Dataset EV5

## Data Availability

Data generated from chemical screens, molecular docking simulations and analyses, and enzymatic inhibition assays are available as Datasets [Link], [Link]. The enzymatic inhibition assay results have also been deposited on BioStudies (https://www.ebi.ac.uk/biostudies/studies/S‐BSST863?key=082576e6‐3bd2‐4589‐9640‐f04b8092f5cb) to improve accessibility and provide a benchmarking dataset for antibiotic‐protein‐ligand interactions.
