# Supplementary material for: Benchmarking AlphaFold‐enabled molecular docking predictions for antibiotic discovery
Source: Mol Syst Biol. 2022 Sep 6;18(9):e11081. doi: 10.15252/msb.202211081 (PMC9446081; doi:10.15252/msb.202211081)
Supplement: Supplementary file 1 — Expanded View Figures PDF [file MSB-18-e11081-s002.pdf]

Expanded View Figures

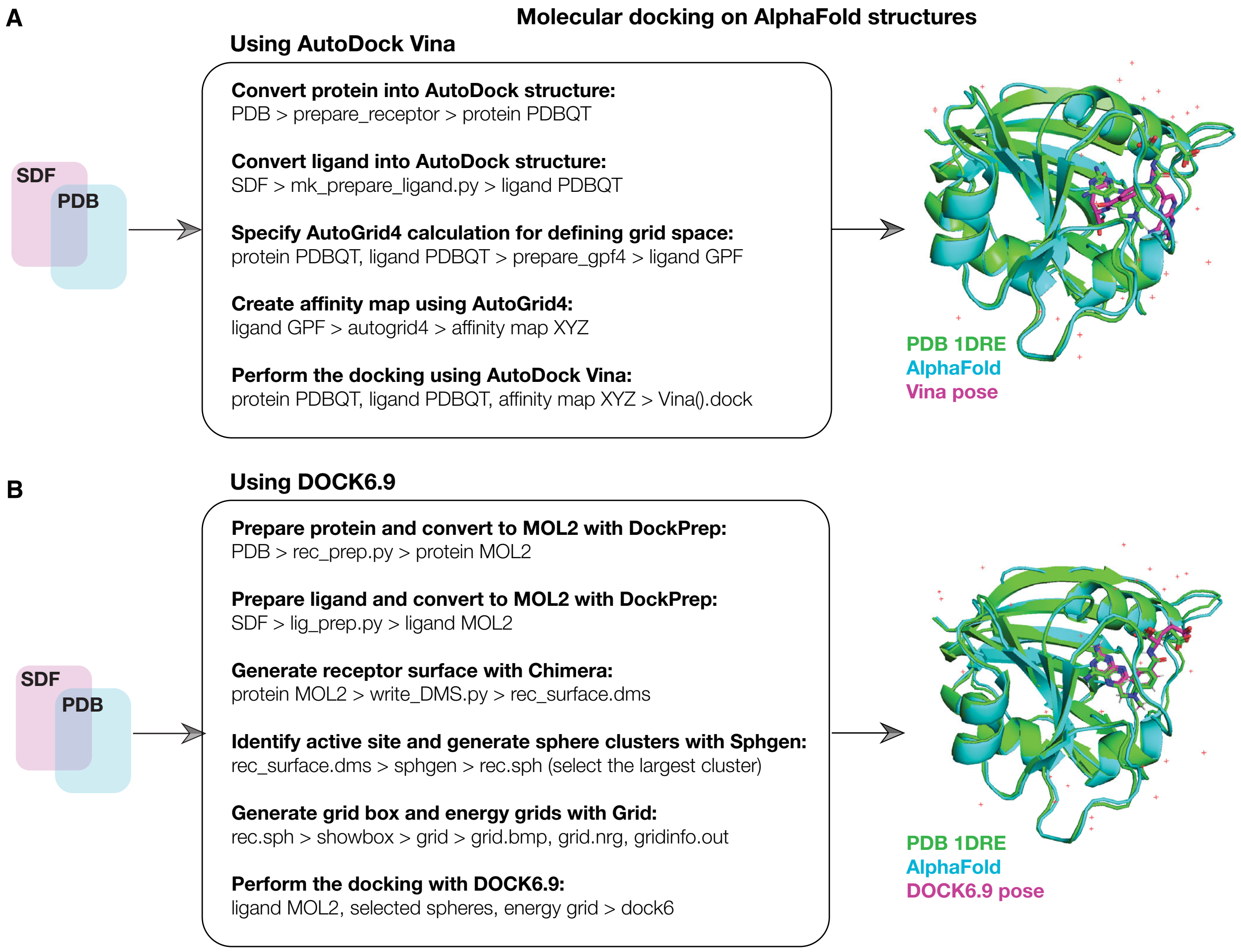

Figure EV1. Schematic of the computational docking approach.

A (Left) Schematic of the computational docking approach using AutoDock Vina. 296 essential proteins in *E. coli* were identified, and their AlphaFold2-predicted structures were curated. The 218 active compounds and 100 inactive compounds were represented in three dimensions in SDF files. All compounds and proteins were prepared for docking as shown and then docked using AutoDock Vina run on a high-performance computing server. The resulting binding pose and thermodynamic binding affinity predictions for all 64,528 (active compounds) and 29,600 (inactive compounds) pairwise protein-ligand interactions were analyzed and ranked. (Right) Superimposed predicted and experimental structures for methotrexate binding to *E. coli* FolA (PDB 1DRE), which was used as a positive docking control from the Protein Data Bank (Dataset EV2).

B Similar to (A), but for the docking of 218 active compounds and 100 inactive compounds, and a subset of 12 essential proteins, respectively, using DOCK6.9. The 12 selected essential proteins correspond to all proteins empirically tested in this study.

© 2022 The Authors

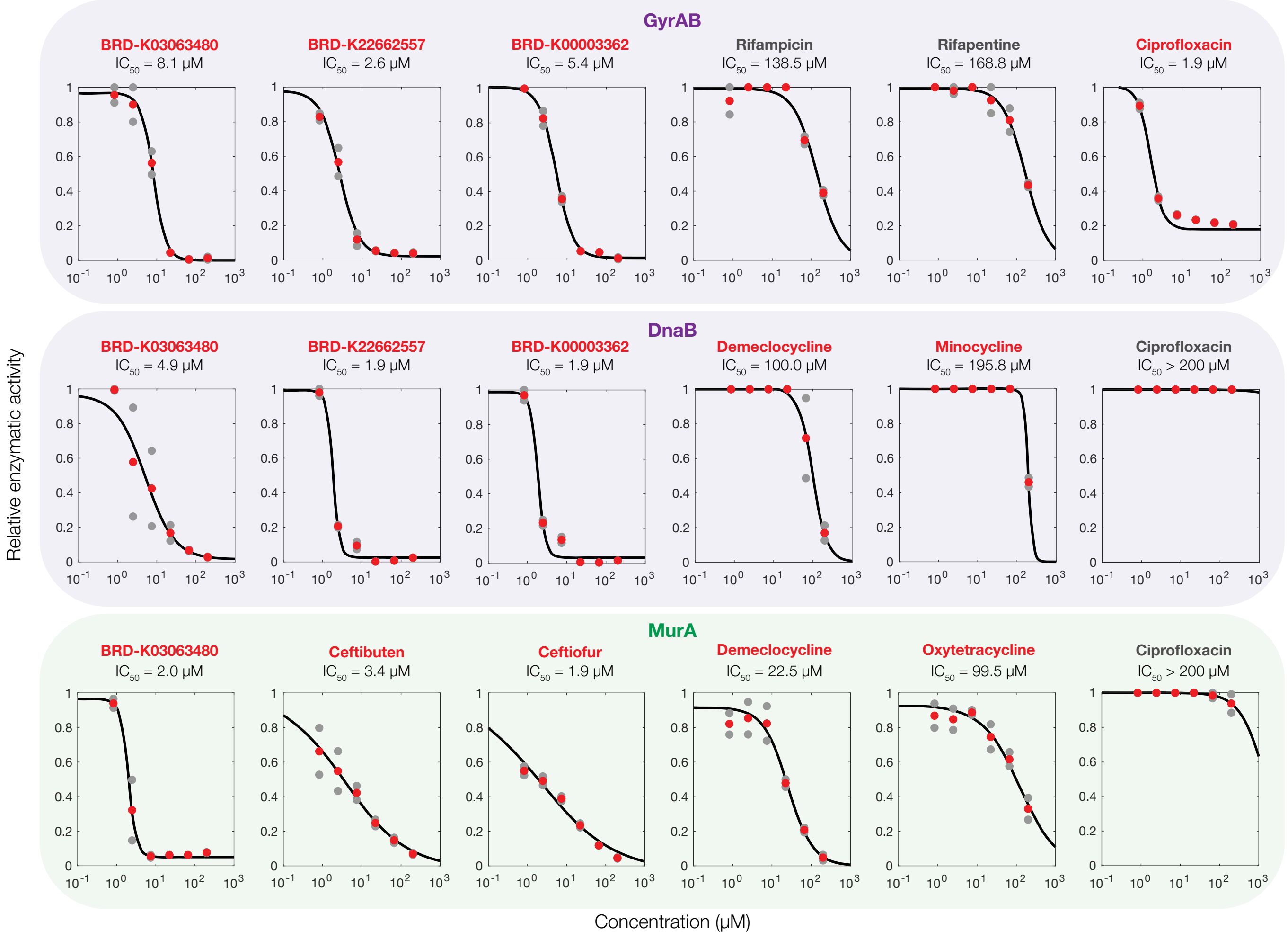

Figure EV2. Enzymatic inhibition screen validation experiments.

Dose–response experiments showing the relative enzymatic activity of three essential proteins, each treated with six antibacterial compounds. Relative activity is measured with respect to untreated controls. Data from two biological replicates (gray points) are shown, and mean activity values (red points) were fit to Hill functions (black curves) to estimate IC_{50} values. Compound names in red indicate hits from the screens shown in Fig 3 of the main text, and compound names in gray indicate non-hits. Essential proteins correspond to the genes indicated and are involved in DNA replication (purple) and cell wall synthesis (green).

 © 2022 The Authors

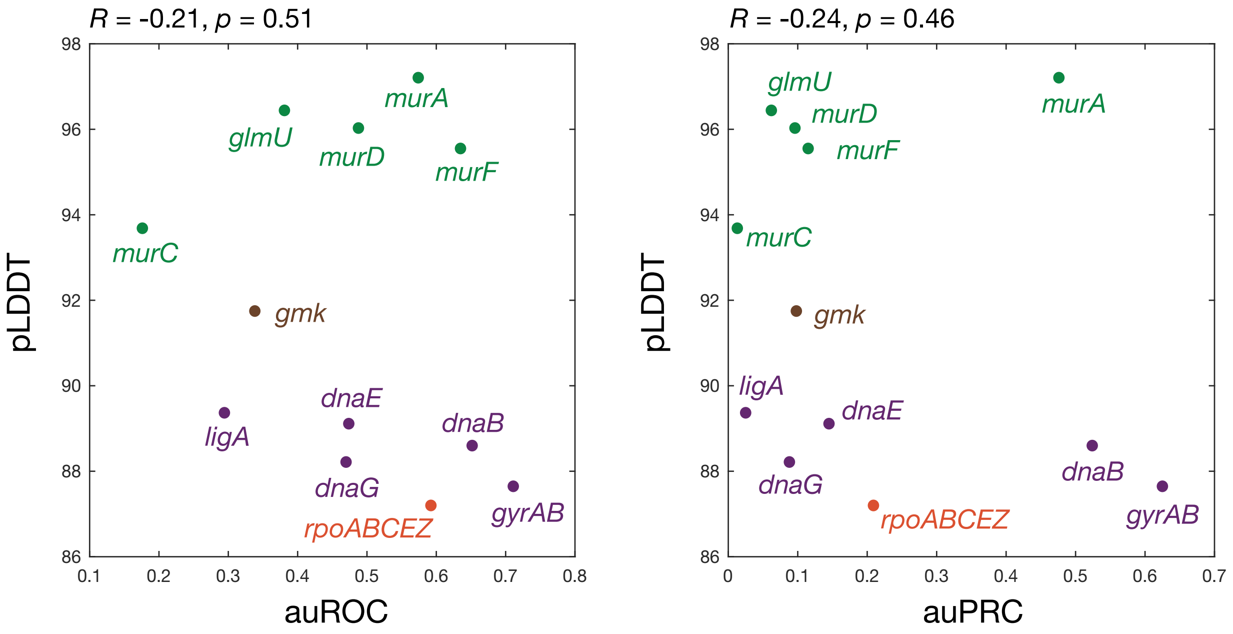

Figure EV3. No correlation between model performance and AlphaFold2 prediction confidence.

Shown is a plot of the auROC values from Fig 4D of the main text or auPRC values from Fig EV5 against AlphaFold2's per-residue confidence score (pLDDT), averaged across each protein, for all 12 empirically tested essential proteins. Essential proteins correspond to the genes indicated and are involved in DNA replication (purple), transcription (orange), metabolism (brown), and cell wall synthesis (green). Higher pLDDT scores indicate higher model confidence. The Pearson correlation coefficients, *R*, and corresponding *P*-values are indicated.

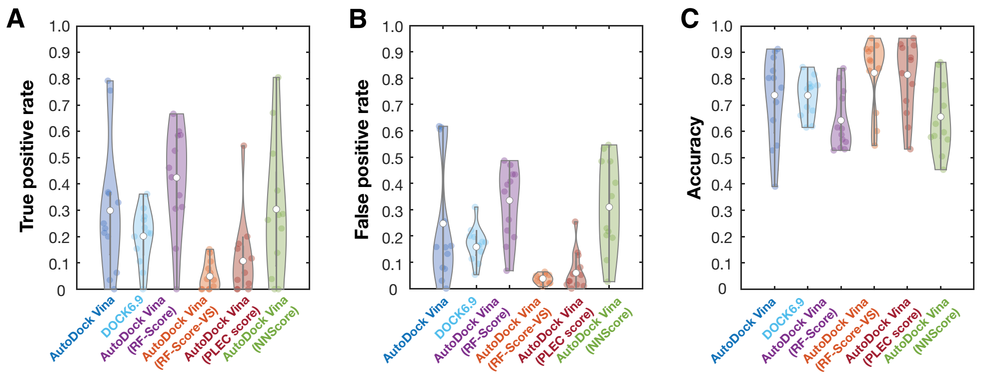

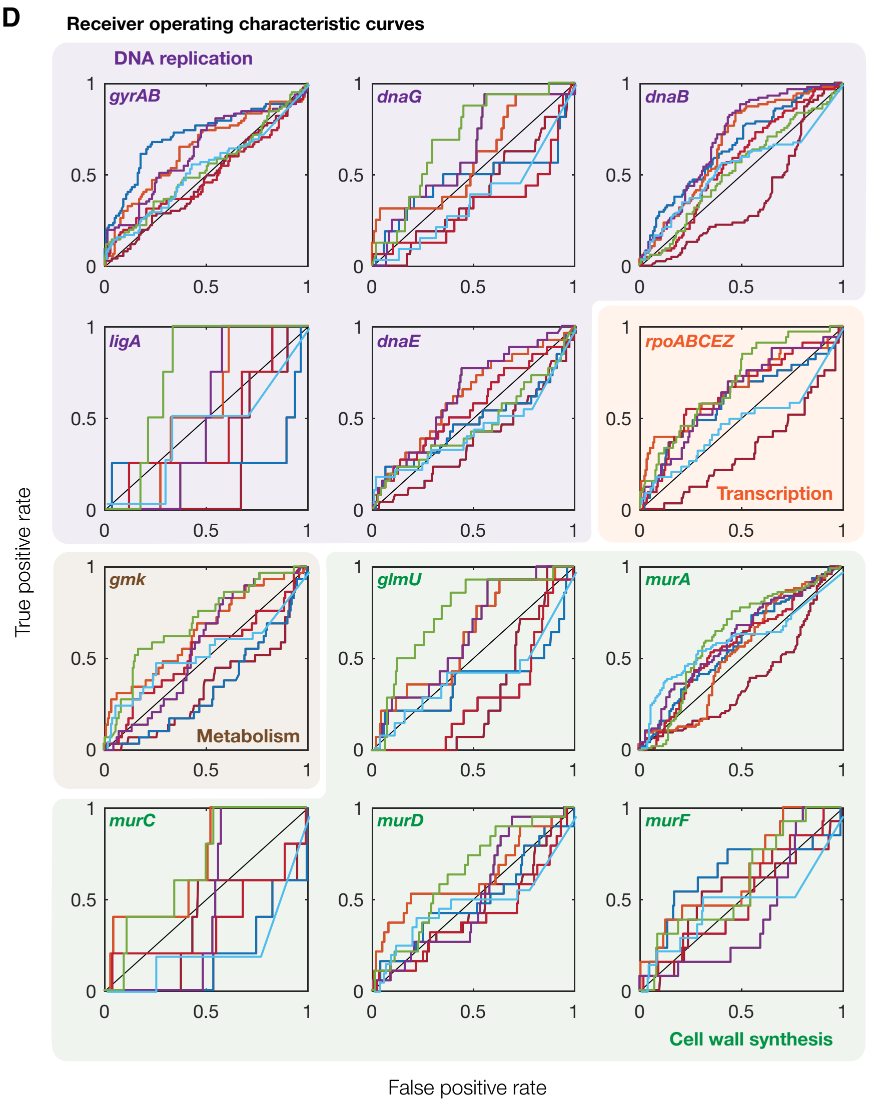

E

auROCs

	AutoDock Vina	DOCK6.9	AutoDock Vina (RF-Score)	AutoDock Vina (RF-Score-VS)	AutoDock Vina (PLEC score)	AutoDock Vina (NNScore)
gyrAB	0.711	0.519	0.635	0.527	0.485	0.628
dnaG	0.470	0.355	0.584	0.701	0.329	0.626
dnaB	0.651	0.528	0.660	0.523	0.561	0.691
ligA	0.294	0.422	0.555	0.750	0.445	0.511
dnaE	0.474	0.426	0.621	0.461	0.556	0.635
rpoABCEZ	0.592	0.458	0.660	0.693	0.634	0.629
gmk	0.338	0.546	0.645	0.698	0.524	0.565
glmU	0.381	0.420	0.618	0.746	0.279	0.627
murA	0.574	0.610	0.527	0.619	0.565	0.607
murC	0.176	0.245	0.693	0.680	0.366	0.457
murD	0.488	0.503	0.617	0.632	0.398	0.514
murF	0.635	0.498	0.629	0.582	0.502	0.406
**Average**	**0.482**	**0.461**	**0.620**	**0.634**	**0.470**	**0.575**

Figure EV4.

© 2022 The Authors

Figure EV4. Model performance for different rescoring functions.

A–C Distributions of true-positive rates (A), false-positive rates (B), and accuracy (C) across all 12 empirically tested essential proteins, for binding affinity thresholds of −7 kcal/mol (AutoDock Vina), −70 kcal/mol (DOCK6.9), or $pK_d > 7$ (AutoDock Vina with all rescoring functions). White points indicate mean values, and gray bars indicate ranges of 25th to 75th percentile values (Q_1 and Q_3, respectively). The whiskers of the gray box plots indicate ranges of values not considered outliers, that is, those between $Q_1 - 1.5 \times$ IQR and $Q_3 + 1.5 \times$ IQR, where IQR $= Q_3 - Q_1$ is the interquartile range.

D Receiver operating characteristic (ROC) curves for all 12 empirically tested essential proteins. Essential proteins correspond to the genes indicated and are involved in DNA replication (purple), transcription (orange), metabolism (brown), and cell wall synthesis (green). The black diagonal line indicates the benchmark generated by random guessing. ROC curves are colored according to the model used. Curves for AutoDock Vina (dark blue curves) are identical to those shown in Fig 4D of the main text and are reproduced here to facilitate comparison between models.

E Area under the ROC curve (auROC) values for each empirically tested essential protein and each model used.

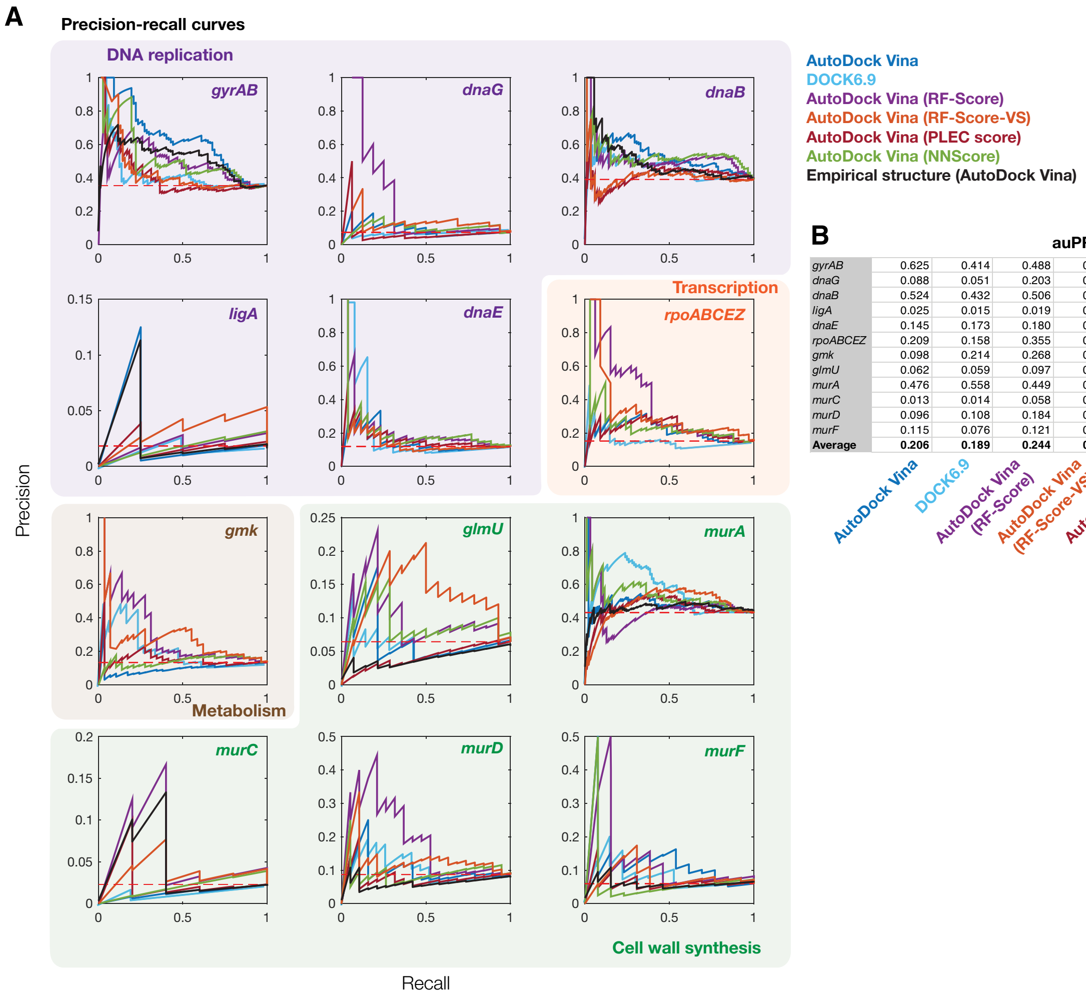

B

auPRCs

	AutoDock Vina	DOCK6.9	AutoDock Vina (RF-Score)	AutoDock Vina (RF-Score-VS)	AutoDock Vina (PLEC score)	AutoDock Vina (NNScore)	Empirical structure (AutoDock Vina)	Baseline hit fraction
gyrAB	0.625	0.414	0.488	0.437	0.402	0.504	0.556	0.353
dnaG	0.088	0.051	0.203	0.121	0.066	0.100	N/A	0.073
dnaB	0.524	0.432	0.506	0.397	0.409	0.522	0.483	0.390
ligA	0.025	0.015	0.019	0.033	0.017	0.017	0.030	0.018
dnaE	0.145	0.173	0.180	0.127	0.161	0.170	N/A	0.119
rpoABCEZ	0.209	0.158	0.355	0.304	0.219	0.221	N/A	0.151
gmk	0.098	0.214	0.268	0.242	0.151	0.140	N/A	0.133
glmU	0.062	0.059	0.097	0.131	0.041	0.089	0.040	0.064
murA	0.476	0.558	0.449	0.477	0.455	0.520	0.447	0.431
murC	0.013	0.014	0.058	0.037	0.024	0.020	0.049	0.023
murD	0.096	0.108	0.184	0.124	0.076	0.088	0.067	0.087
murF	0.115	0.076	0.121	0.078	0.062	0.063	0.053	0.060
**Average**	**0.206**	**0.189**	**0.244**	**0.209**	**0.174**	**0.205**	**0.216**	**0.159**

Figure EV5. Precision-recall for different rescoring functions.

A Precision-recall (PR) curves for all 12 empirically tested essential proteins. Essential proteins correspond to the genes indicated and are involved in DNA replication (purple), transcription (orange), metabolism (brown), and cell wall synthesis (green). The red dashed line indicates the benchmark generated by random guessing, corresponding to recall values equal to the baseline hit fraction. PR curves are colored according to the model or protein structure used.

B Area under the PR curve (auPRC) values for each empirically tested essential protein and each model used.
