# Supplementary material for: Benchmarking AlphaFold‐enabled molecular docking predictions for antibiotic discovery
Source: Mol Syst Biol. 2022 Sep 6;18(9):e11081. doi: 10.15252/msb.202211081 (PMC9446081; doi:10.15252/msb.202211081)
Supplement: Supplementary file 8 [file MSB-18-e11081-s003.pdf]

molecular systems biology

Benchmarking AlphaFold-enabled molecular docking predictions for antibiotic discovery

Felix Wong, Aarti Krishnan, Erica Zheng, Hannes Stärk, Abigail Manson, Ashlee M. Earl, Tommi Jaakkola, and James Collins
DOI: 10.15252/msb.202211081

Corresponding author(s): James Collins (jimjc@mit.edu)

Review Timeline:

Submission Date:	18th Apr 22
Editorial Decision:	20th May 22
Revision Received:	24th Jun 22
Accepted:	26th Jul 22

Editor: Maria Polychronidou

Transaction Report:

(Note: With the exception of the correction of typographical or spelling errors that could be a source of ambiguity, letters and reports are not edited. Depending on transfer agreements, referee reports obtained elsewhere may or may not be included in this compilation. Referee reports are anonymous unless the Referee chooses to sign their reports.)

Thank you again for submitting your work to Molecular Systems Biology. We have now heard back from the three reviewers who agreed to evaluate your study. As you will see below, the reviewers think that the study seems potentially interesting. However, they raise a series of concerns, which we would ask you to address in a revision.

I think that the reviewers' recommendations are rather clear and I therefore see no need to repeat the comments listed below. One important issue refers to the need to strengthen the conclusions related to the machine-learning-based scoring functions. All issues raised by the referees would need to be satisfactorily addressed. Please let me know in case you would like to discuss in further detail any of the issues raised, I would be happy to schedule a call.

On a more editorial level, we would ask you to address the following points:

Reviewer #1:

Although molecular docking provides an important tool to computationally identify protein-ligand interactions and drug mechanisms of action, the availability of 3D structures of proteins has traditionally been a bottleneck for researchers using docking. Enabled by the emergence of deep learning-based protein 3D structure, prediction methods now make it possible to perform large-scale structure-based protein-ligand interaction identification campaigns.
In this manuscript, the authors analyzed the protein-ligand binding prediction obtained from molecular docking with AlphaFold2-predicted protein structures. By comparing the protein-ligand binding predictions to experimentally determined enzymatic inhibition data between 12 essential E. coli proteins and 218 active antibacterial compounds, the authors demonstrated that (1) prediction performance of molecular docking using AlphaFold2-predicted protein structures is similar to that of using experimentally determined structures, (2) molecular docking alone can only show predictive ability for protein-ligand interaction identification on a subset of proteins, and (3) machine learning-based protein-ligand binding scoring for molecular docking poses demonstrated predictive power for protein-ligand interaction identification (i.e., AUROC > 0.5) on the majority of the 12 tested proteins. The manuscript demonstrated the feasibility of using AlphaFold2-predicted protein structures to address the structure availability issue in molecular docking and highlighted the limitation of current protein-ligand scoring methods and the need to develop machine learning-based approaches to improve scoring of docking-based protein-ligand interaction. In addition, an experimentally validated protein-ligand affinity dataset for 218 antibacterial compounds and 12 E. coli proteins, and 142 antibiotic-protein target pairs previously reported in the literature, were generated in this manuscript as benchmark datasets for future studies. Overall, the manuscript is well-written, and the data presented convincing. The following minor issues need to be addressed:

(1) Docking study for inactive compounds. The docking study in the manuscript covered 218 compounds that are experimentally active against E. coli. It would be interesting to also study inactive compounds that would serve as negative controls. For example, the authors could randomly select inactive compounds from the screened library and perform docking studies to see (a) if these compounds can be docked to the binding sites, and (b) check and compare the binding scores of these inactive compounds to those of active compounds. In the case that a large number of inactive compounds can be docked into the different binding sites with high binding scores, the authors would need to address potential false positive rate issues.
(2) The authors used two open-source docking programs in the manuscript. The authors should comment on how using other software to perform docking and scoring, such as Schrödinger, would influence the results.

Reviewer #2:

Wong et al. present an integrated experimental-computational study to perform high-throughput molecular docking on bacterial proteins making use of alphafold predictions (as well as experimental structures). After screening 40k compounds in a phenotypic screen of E.coli survival, they docked the 218 compounds found to be active against a set of 296 proteins thought to be essential, and recovered a number of known antibiotic-target interactions. They then performed dedicated enzymatic activity inhibition assays and benchmarked their molecular docking results against these assays finding that they are roughly as good as random, both based on alphafold structure, as well as on experimental ones. They also constructed a set of meta-predictors which they find to perform somewhat better.
I find this to be a somewhat eclectic study. First, the "benchmarking of docking on alphafold structures" really is only done on 8 proteins (the only ones where a comparison was made to crystal structures), and even there, the results aren't conclusive; since the results are found to be basically random for crystal structures, really no conclusion can be derived whether alphafold structure are reliable for docking. Second, much work has been done on benchmarking of different virtual screening protocols and scoring functions (though I'm no expert in this direct area), so the addition to this subfield here is largely an evaluation of testing on 12 targets.
On the other hand, they do provide a complete study, from high-throughput screening to dedicated biochemical inhibition experiments, as well as molecular docking and evaluation of a number of ML-based scoring functions, so the comprehensiveness and amount of data provided should be commended.
Major points:

1) I find it likely that the authors aren't experts in machine learning or related fields. For instance, they rely heavily on AU-ROC, which in such cases with a large imbalance (that I'm assuming is the case here) between negative and positive data is usually a problematic measure. Also, their "comparison of model predictions with known antibiotic binding targets" isn't very meaningful (just knowing the true positive rates without knowing anything about false positives doesn't give any information about the classifier). It also seems likely to me that their "wisdom of crowds" approach is just a classic case of overfitting. In any case, I would warmly recommend that the authors recruit a proper expert in this field to help out.
2) It has been recognized that molecular docking on crystal structures isn't the best way forward, and I'm aware that real experts to a fair bit of preprocessing of the structures using e.g., long molecular dynamics simulations to identify pockets. Surely this would affect the authors' results.

Reviewer #3:

In this paper, the authors sought to test whether AlphaFold2 protein structure predictions could be used for reverse docking, that

is, prediction of binding targets of antibacterial compounds. They identify 218 compounds with anti-bacterial activity, which they seek to dock to 296 AlphaFold2-predicted essential protein structures. These predictions seemed to suggest widespread compound and protein promiscuity, which they experimentally validate using enzymatic inhibition assays. Using the results of these assays and published interactions, they however find that the accuracy of the reverse docking predictions is poor (auROC~0.5 on average). They do find that molecular docking using AlphaFold2-predicted structures provides similar performance compared to using experimentally determined structures. For AlphaFold2 structures, using machine learning-based scoring functions for docking in some cases improves the accuracy.

Overall this is an interesting idea but the conclusions are largely negative, and the main conclusion is that reverse docking as currently done provides poor performance. The contribution of AlphaFold2 predictions to this study is moderately clear.

One important technical aspect that is missing is confidence intervals for auROC. It is very hard to compare auROC values without such confidence intervals, and without them, one cannot conclude that one auROC value, eg obtained by ML based scoring functions is higher than another one. This needs to be addressed.

Wong*, Krishnan*, Zheng, Stärk, Manson, Earl, Jaakkola, and Collins, "Benchmarking AlphaFold-enabled molecular docking predictions for antibiotic discovery"

Summary of main changes.

We thank all the reviewers for their constructive and thoughtful comments on the paper. We have addressed all of the points raised by the reviewers through additional experiments, analyses, and revisions, which have significantly strengthened the work. We would like to highlight the following key additions and revisions to the paper:

(1) To address Reviewers #1 and #2's comments on false positives, we have performed additional docking simulations with 100 compounds that are inactive against (i.e., do not inhibit the growth of) *E. coli.* The results are now presented in Fig. 2 and included in Dataset EV2. We found that all the sampled inactive compounds could be docked to the predicted binding sites of the 296 essential proteins, and intriguingly, that the binding scores of these inactive compounds are largely similar to those of the active compounds. We believe that this result further highlights one of the main messages of the present work, which is that the performance of molecular docking in identifying true protein-ligand binding pairs is weak. We have described these results in detail on lines 152-169 of the main text and discussed that docking is known to produce many false positives there. As a result, we believe that these points motivate a more detailed analysis of the docking predictions for active compounds, which could determine whether there is still any predictive power (indeed, one of the main points of this study is that there largely is not).

(2) To address Reviewer #1's comments on other docking and scoring software, we have now further discussed how using other software would influence our results, and we have pointed out that prior benchmarking studies using Schrödinger's Glide and other software on the directory of useful decoys (DUD) dataset suggest that the accuracy of our docking predictions would remain largely similar (Durrant et al, 2013; Pereira et al, 2016) on lines 394-399 of the revised paper.

(3) To address Reviewer #2's comments on the potential class imbalance of our protein-ligand interaction data, we have generated precision-recall (PR) curves and calculated area under the PR curve (auPRC) values for each protein (Fig. EV5). To address Reviewer #2's comments on potential overfitting of machine learning models, we have better detailed the minimal overlap between our model training and test sets (*Methods*). We have consulted Professor Tommi Jakkola and Mr. Hannes Stärk for their input and help on responding to this point on potential overfitting and all other aspects of the machine learning approaches used in this work. Due to these important contributions, we have now added Prof. Jakkola and Mr. Stärk as co-authors of this manuscript.

(4) As suggested by Reviewer #3, we have provided 95% confidence interval information for all auROC (and auPRC) values in Table EV1. We have revised the main text and Discussion to further emphasize the main message of our work, which is that improvements in molecular docking—for instance, using machine learning—are needed to fully leverage AlphaFold's structural information. We believe that this conclusion is especially timely in light of the excitement from AlphaFold and its implications for drug discovery.

In the following, line numbers and citations refer to the revised version of the paper, and responses are indicated in blue font.

Reviewer #1.

We thank the reviewer for their thoughtful and enthusiastic comments.

1. **Although molecular docking provides an important tool to computationally identify protein-ligand interactions and drug mechanisms of action, the availability of 3D structures of proteins has traditionally been a bottleneck for researchers using docking. Enabled by the emergence of deep learning-based protein 3D structure, prediction methods now make it possible to perform large-scale structure-based protein-ligand interaction identification campaigns.**

   In this manuscript, the authors analyzed the protein-ligand binding prediction obtained from molecular docking with AlphaFold2-predicted protein structures. By comparing the protein-ligand binding predictions to experimentally determined enzymatic inhibition data between 12 essential E. coli proteins and 218 active antibacterial compounds, the authors demonstrated that (1) prediction performance of molecular docking using AlphaFold2-predicted protein structures is similar to that of using experimentally determined structures, (2) molecular docking alone can only show predictive ability for protein-ligand interaction identification on a subset of proteins, and (3) machine learning-based protein-ligand binding scoring for molecular docking poses demonstrated predictive power for protein-ligand interaction identification (i.e., AUROC > 0.5) on the majority of the 12 tested proteins. The manuscript demonstrated the feasibility of using AlphaFold2-predicted protein structures to address the structure availability issue in molecular docking and highlighted the limitation of current protein-ligand scoring methods and the need to develop machine learning-based approaches to improve scoring of docking-based protein-ligand interaction. In addition, an experimentally validated protein-ligand affinity dataset for 218 antibacterial compounds and 12 E. coli proteins, and 142 antibiotic-protein target pairs previously reported in the literature, were generated in this manuscript as benchmark datasets for future studies. Overall, the manuscript is well-written, and the data presented convincing.

   We thank the reviewer for their appreciation of our work, and we hope that our revisions and responses provided below sufficiently address their comments.

2. **(1) Docking study for inactive compounds. The docking study in the manuscript covered 218 compounds that are experimentally active against E. coli. It would be interesting to also study inactive compounds that would serve as negative controls. For example, the authors could randomly select inactive compounds from the screened library and perform docking studies to see (a) if these compounds can be docked to the binding sites, and (b) check and compare the binding scores of these inactive compounds to those of active compounds. In the case that a large number of inactive compounds can be docked into the different binding sites with high binding scores, the authors would need to address potential false positive rate issues.**

   We thank the reviewer for this important comment and agree. To address this point, we have repeated our docking simulations with 100 randomly selected compounds that are inactive against (do not inhibit the growth of) *E. coli.* The results are now presented in Fig. 2 and included in Dataset EV2. We found that all the sampled inactive compounds could be docked to the binding sites of the 296 essential proteins, and intriguingly, that the binding scores of these inactive compounds are largely similar to those of the active compounds. We believe that this result further highlights one of the main messages of the present work, which is that the performance of molecular docking in identifying true protein-ligand binding pairs is weak. We have described these results in detail on lines 151-168 of the main text and discussed that docking is known to produce many false positives there. As a result, we believe that these points motivate a more detailed analysis of the docking predictions for active compounds, which could determine whether there is still any predictive power (indeed, one of the main points of this study is that there largely is not).

3. **(2) The authors used two open-source docking programs in the manuscript. The authors should comment on how using other software to perform docking and scoring, such as Schrödinger, would influence the results.**

   We thank the reviewer for pointing this out. Although we have focused on using AutoDock Vina and DOCK6.9 in this study as commonly used and popular docking platforms, we agree that other software for performing docking and scoring could influence some of our detailed protein-ligand binding predictions.

Nevertheless, we believe that prior benchmarking studies using Schrödinger's Glide and other software on the directory of useful decoys (DUD) dataset suggest that the accuracy of our docking predictions would remain largely similar (Durrant et al, 2013; Pereira et al, 2016). On lines 394-399 of the revised paper, we have now discussed how using other software would influence our results, and we have pointed out studies that have exhaustively benchmarked an array of docking and scoring software on previously available datasets.

We thank the reviewer for thoughtfully pointing out this and all other points, which have helped us to significantly improve our work.

Reviewer #2.

We thank the reviewer for their insightful and detailed report.

4. **Wong et al. present an integrated experimental-computational study to perform high-throughput molecular docking on bacterial proteins making use of alphafold predictions (as well as experimental structures). After screening 40k compounds in a phenotypic screen of E.coli survival, they docked the 218 compounds found to be active against a set of 296 proteins thought to be essential, and recovered a number of known antibiotic-target interactions. They then performed dedicated enzymatic activity inhibition assays and benchmarked their molecular docking results against these assays finding that they are roughly as good as random, both based on alphafold structure, as well as on experimental ones. They also constructed a set of meta-predictors which they find to perform somewhat better.**

We thank the reviewer for their appreciative and insightful comments, which have helped to strengthen the piece considerably, and we hope that the reviewer finds their concerns adequately addressed with our introduced revisions and responses described below.

5. **I find this to be a somewhat eclectic study. First, the "benchmarking of docking on alphafold structures" really is only done on 8 proteins (the only ones where a comparison was made to crystal structures), and even there, the results aren't conclusive; since the results are found to be basically random for crystal structures, really no conclusion can be derived whether alphafold structure are reliable for docking.**

We agree with the reviewer that the comparison between docking predictions using AlphaFold structures and those using empirically evidenced crystal structures is for eight proteins (blue curves in Fig. 4D, and black curves in Fig. EV5). Here, the reviewer correctly points out that we found the docking predictions using empirically evidenced crystal structures to be largely similar to those using AlphaFold structures, suggesting that the weak docking performance may not caused by AlphaFold's limitations. These points are discussed in detail on lines 288-298 of the revised paper. However, we also would like to respectfully point out that we have not focused on evaluating the quality of AlphaFold structures in this work. Rather, we have aimed to determine whether a commonly used, reverse-docking approach to predicting protein-ligand interactions performs well given AlphaFold structures—a question which could inform our current abilities to make use of general structures given by AlphaFold. We have sought to clarify this point by rewriting lines 298-303 and lines 384-388 of the paper, and we apologize to the reviewer for not making this motivation clear previously.

More generally, we agree that the number of essential proteins (12) evaluated in our study is substantially less than the total number of essential proteins in *E. coli* (296). We believe that data measuring direct protein-ligand binding activity are necessarily scarce and hard-to-obtain, as only a handful of methods exist for probing protein-ligand binding interactions (e.g., enzymatic activity assays, differential scanning fluorimetry, and surface plasmon resonance). Of these methods, only a few can be made high-throughput. We have resorted to high-throughput enzymatic activity assays in our study and have performed these assays for all proteins for which we could readily do so. Accordingly, we believe that our dataset is both unique—measuring both binders and non-binders—and will inspire future studies that use more diverse methods to

further assess protein-ligand binding interactions. In order to make these points clearer to all readers, we have revised lines 440-451 in the Discussion, which also calls for future work to generate more original protein-ligand binding datasets.

6. **Second, much work has been done on benchmarking of different virtual screening protocols and scoring functions (though I'm no expert in this direct area), so the addition to this subfield here is largely an evaluation of testing on 12 targets.**

We thank the reviewer for this important comment and agree that there has been much work on benchmarking different docking and scoring software. We have revised lines 394-399 to mention this literature, and we have revised lines 440-451 to better contextualize the dataset contributions of the present work. Notably, we wish to point out that our work differs from other studies in two important ways: (1) we have based our docking simulations on AlphaFold structures; and (2) we have generated our own dataset, using enzymatic activity assays for 12 essential *E. coli* proteins, that include both binders and non-binders to any given protein. While typical benchmarking studies (Durrant et al, 2013; Pereira et al, 2016) have employed well-studied (and often used) crystal structures, we believe that the first point is important because our results suggest that further work in improving docking is needed to better leverage the protein structures predicted by AlphaFold. We feel that this point is especially timely in light of the excitement from AlphaFold and its implications for drug discovery. Additionally, typical benchmarking studies have not generated their own datasets and have instead relied on well-studied (and often used) datasets like DUD-E (Mysinger et al, 2012). These datasets were compiled by amalgamating a large number of studies and crystal structures known to form complexes, and may therefore not be well-controlled; indeed, recent studies have suggested a substantial degree of hidden bias in DUD-E, which might bias docking predictions (Chen et al, 2019). More importantly, DUD-E contains only 102 protein targets that are mostly specific to *Homo sapiens* (Mysinger et al, 2012), and only three proteins—beta-lactamase, peptide deformylase, and thymidylate synthase, none of which we study here—from *E. coli.* Evidently, new datasets and benchmarks for more diverse organisms, like *E. coli*, are needed for antibiotic discovery. We believe that the present study contributes to this important task and have made our dataset publicly available on BioStudies (https://www.ebi.ac.uk/biostudies/studies/S-BSST863?key=082576e6-3bd2-4589-9640-f04b8092f5cb), which we hope will inspire further related work.

7. **On the other hand, they do provide a complete study, from high-throughput screening to dedicated biochemical inhibition experiments, as well as molecular docking and evaluation of a number of ML-based scoring functions, so the comprehensiveness and amount of data provided should be commended.**

We thank the reviewer for their appreciation of this work. We very much agree that it is important to generate original datasets to test docking predictions, and we hope that our work has provided a comprehensive and inspiring evaluation of different docking and scoring approaches in order to determine whether we might be able to fully leverage AlphaFold protein structures for the identification of true protein-ligand interactions.

8. **Major points: 1) I find it likely that the authors aren't experts in machine learning or related fields. For instance, they rely heavily on AU-ROC, which in such cases with a large imbalance (that I'm assuming is the case here) between negative and positive data is usually a problematic measure. Also, their "comparison of model predictions with known antibiotic binding targets" isn't very meaningful (just knowing the true positive rates without knowing anything about false positives doesn't give any information about the classifier). It also seems likely to me that their "wisdom of crowds" approach is just a classic case of overfitting. In any case, I would warmly recommend that the authors recruit a proper expert in this field to help out.**

We apologize for previously being unclear. We had chosen to present ROC curves and assess prediction accuracy using the auROC because our experimental results did not generally indicate a large class imbalance between hits and non-hits. Fig. 3B shows the distribution of empirically validated hits: while two proteins, *murC* and *ligA*, had only 5 and 4 hits, respectively, all other proteins had at least 13 hits, and three proteins (*gyrA, murA,* and *dnaB*) had more than 70 (>30% of all tested active compounds). Nevertheless, we agree with the reviewer that presenting assessments that better control for class imbalance could strengthen the

quality of our analysis. We have therefore generated precision-recall (PR) curves and calculated area under the PR curve (auPRC) values for each protein. We have summarized the statistics of the auPRC values on lines 273-285, lines 297-298, and lines 338-339 of the revised paper, and presented the PR curves in Fig. EV5. Accompanying 95% confidence interval estimates for all auROC and auPRC values are now provided in Table EV1.

We also thank the reviewer for their comment on true positive rates and agree. We had mainly intended for our discussion on known antibiotic binding targets to inform a reasonable binding affinity threshold for the docking predictions, and using this to assess the performance of the classifier would indeed require knowledge about false positives. To address this comment, we have now appended a discussion of corresponding false positive rates and the implications on model performance on lines 180-186 of the revised paper. Additionally, in response to a comment made by Reviewer #1, we have repeated our docking simulations with 100 compounds that are inactive against (i.e., do not inhibit the growth of) *E. coli*. The results are now presented in Fig. 2 and included in Dataset EV2. We found that all the sampled inactive compounds could be docked to the predicted binding sites of the 296 essential proteins, and intriguingly, that the predicted binding affinities of these inactive compounds are largely similar to those of the active compounds. We believe that this result further highlights one of the main messages of the present work, which is that the performance of molecular docking in identifying true protein-ligand binding pairs is weak. We have described these results in detail on lines 152-169 of the main text and mentioned that docking is known to produce many false positives there. Additionally, in relation to the discussion on false positive rates, we have revised lines 184-191 to read: "If true protein-ligand interactions were rare, this would suggest that the false positive rates predicted by our model are comparable to its true positive rates, even for a stringent binding affinity threshold of -7 kcal/mol. Consistent with this reasoning, the same binding affinity thresholds encompass 10% (-7 kcal/mol) and 30% (-5 kcal/mol) of the modeled protein-ligand interactions involving inactive compounds (Fig. 2C), which are likely to not bind any essential protein given that they do not inhibit bacterial growth. This comparison therefore suggests that the performance of our modeling platform is weak." We believe that these additions make our comparison of model predictions with known antibiotic binding targets more meaningful.

In regards to the reviewer's comment that our wisdom of crowds approach may be a case of overfitting, we would like to respectfully point out that ensembling is a common and valid approach in machine learning. An ensemble of models may perform better than any individual model if the regions where the individual models are wrong do not completely overlap.

Of particular relevance to our case, our protein-ligand interaction data shares only one overlapping protein-ligand interaction—rifampicin bound to RNA polymerase (4KMU)—with PDBBind v2016, and none with DUD-E, as mentioned on lines 324-326 of the revised paper. Hence, nearly all the predicted protein-ligand interactions from each model and our ensembling approach pertain to those that the models have not previously seen. Additionally, while there is little protein-ligand interaction overlap between our training and test data, we further investigated the overlap between individual proteins and compounds between the training and test data. We found that the overlap in each case was also minimal, as tabulated below in Table R1.

	Number of overlapping proteins with test set	Number of overlapping ligands with test set	Number of overlapping protein-ligand interactions with test set
PDBBind v2016 (13,308 protein-ligand interactions, 3,095 unique proteins, and 9,642 unique ligands)	**6**<br>*E. coli gyrAB* (2Y3P, 4ZVI), *rpoABCDZ* (4KMU), *gmk* (2F3R), *glmU* (4AA7), *murA* (3ISS), *murD* (2JFH)<br><br>Organisms other than *E. coli*: *ligA* (several bacteria including *S. aureus*), *dnaG* (*Homo sapiens*)	**31**<br>Azidothymidine, Triclosan, Tyfomycine, Trimethoprim, 5-Fluorouracil, Nitroxoline, Aminacrine, Rifampicin, Aztreonam, Piperacillin, Cefoxitin, Cefuroxime, Cefotaxime, Ciprofloxacin, Furazolidone, Bleomycin, Cephalothin, Meropenem, Kanamycin B, Foscarnet, Methylisothiazolinone, Amikacin, Tobramycin, Cefmenoxime,	**1**<br>*rpoABCDEZ* with rifampicin (4KMU)

		Erythromycin A, Azithromycin, Mupirocin, Avibactam, Geneticin, Rifapentine, Chloroxine	
DUD-E (1,434,015 protein-ligand interactions, 102 unique proteins, and 1,200,431 unique ligands)	**0**	**5**<br>Gliotoxin, Zidovudine, Triclosan, Cefditoren pivoxil, Donepezil	**0**

Table R1: Summary statistics for the difference between the training and test sets used in this study. Overlapping compounds were determined by comparing isomeric SMILES or generic ligand names (when available). Where numbers are not equal to zero, all the overlapping proteins, ligands, or protein-ligand interactions are also indicated.

To quantitatively investigate overfitting, we performed additional statistical analyses examining the correlations between the similarity (in terms of protein sequence or chemical structure) of our training and test sets and model performance. For each protein in our training (either PDBbind v2016 or DUD-E) and test set, we curated amino acid sequence information from UniProt. For each pair of proteins between our training and test set, we used BioPython's pairwise2.align.globalxx() function to align the corresponding sequences and determine an alignment score. We quantified the "training set alignment score" of each protein in our test set to the proteins in our training set by taking the maximum alignment score among all proteins in the training set. For proteins with multiple subunits (e.g., *gyrAB*), we further assigned an alignment score to the protein complex by taking the maximum alignment score among the training set alignment scores of each subunit. We then plotted the training set align score values against the per-protein true positive rate and accuracy values for the 8 machine learning-based models shown in Fig. 5C-E (comprising 4 single rescoring models and 4 ensemble models). The results of our correlation analyses are shown below in Fig. R1. We found no statistically significant ($p<0.05$) correlation between protein similarity and model performance in all analyses, demonstrating that our models have not overfitted based on protein similarity.

Next, we performed a similar analysis for ligands, using RDKit to compute the Tanimoto similarity between the 2048-bit Morgan fingerprint representation (ECFP radius of 2) of each pair of ligands in our training and test sets. For each ligand in our test set, we took the maximum Tanimoto similarity score among all ligands in the training set. We then plotted these training set Tanimoto similarity values against the per-protein true positive rate and accuracy values for each of the 8 machine learning-based models shown in Fig. 5C-E (comprising 4 single rescoring models and 4 ensemble models). As before, the results of our correlation analyses are shown below in Fig. R1. We found only 4 statistically significant correlations, and none for our final ensemble of all 5 models. Furthermore, all 4 statistically significant correlations were *negative*, indicating that model performance is anti-correlated with training/test set ligand similarity, and that these may be spurious correlations. These analyses demonstrate that our models have not overfitted based on ligand similarity.

In sum, we believe that the minimal overlap between our training and test sets and our statistical analyses of protein/ligand similarity and model performance provide a robust demonstration of no overfitting. Additionally, we agree with the reviewer that consulting with a machine learning expert would be beneficial for this work. We have therefore consulted Professor Tommi Jakkola and Mr. Hannes Stärk for their input and help on responding to the above point on potential overfitting and all other aspects of the machine learning used in this work. Due to their important contributions, we have now added Prof. Jakkola and Mr. Stärk as co-authors of this manuscript.

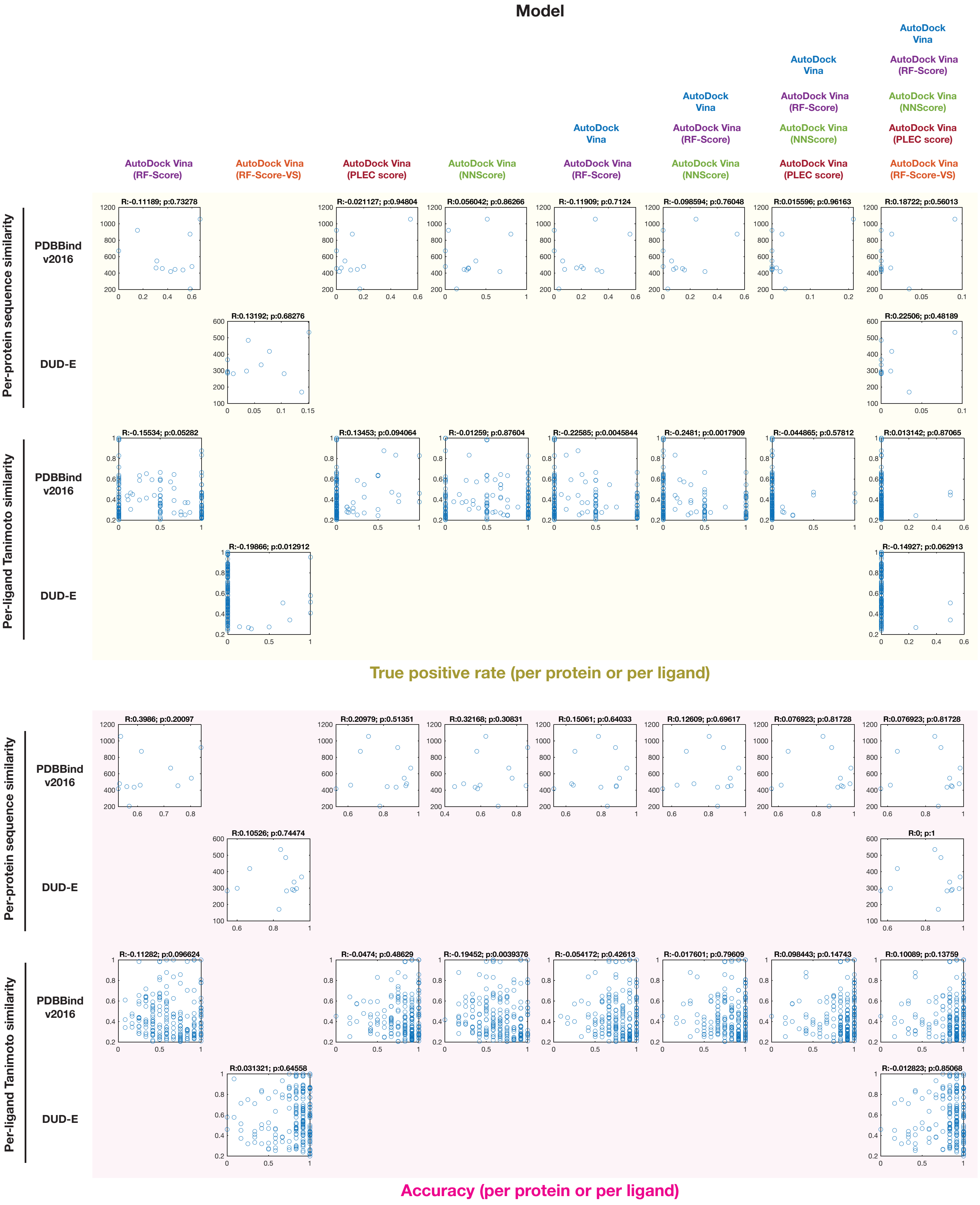

Model
AutoDock Vina (RF-Score)
AutoDock Vina (RF-Score-VS)
AutoDock Vina (PLEC score)
AutoDock Vina (NNScore)
AutoDock Vina
Per-protein sequence similarity
Per-ligand Tanimoto similarity
PDBBind v2016
DUD-E
R:-0.11189; p:0.73278
R:-0.021127; p:0.94804
R:0.056042; p:0.86266
R:-0.11909; p:0.7124
R:-0.098594; p:0.76048
R:0.015596; p:0.96163
R:0.18722; p:0.56013
R:0.13192; p:0.68276
R:0.22506; p:0.48189
R:-0.15534; p:0.05282
R:0.13453; p:0.094064
R:-0.01259; p:0.87604
R:-0.22585; p:0.0045844
R:-0.2481; p:0.0017909
R:-0.044865; p:0.57812
R:0.013142; p:0.87065
R:-0.19866; p:0.012912
R:-0.14927; p:0.062913
True positive rate (per protein or per ligand)
R:0.3986; p:0.20097
R:0.20979; p:0.51351
R:0.32168; p:0.30831
R:0.15061; p:0.64033
R:0.12609; p:0.69617
R:0.076923; p:0.81728
R:0.076923; p:0.81728
R:0.10526; p:0.74474
R:0; p:1
R:-0.11282; p:0.096624
R:-0.0474; p:0.48629
R:-0.19452; p:0.0039376
R:-0.054172; p:0.42613
R:-0.017601; p:0.79609
R:0.098443; p:0.14743
R:0.10089; p:0.13759
R:0.031321; p:0.64558
R:-0.012823; p:0.85068
Accuracy (per protein or per ligand)

Fig. R1: Plots of protein and ligand similarity to the training set and model performance. For a given model (single or ensemble), each plot represents either a per-protein (12 points) or per-ligand (218 points) comparison of the protein or ligand similarity to all proteins or ligands in either PDBbind v2016 or DUD-E, depending on which set was used for training. Model performance is assessed using either true positive rate or accuracy. In each plot, the Spearman's correlation coefficient (*R*) and *p*-value (*p*) are indicated. Overlapping proteins have high sequence similarity, and overlapping ligands have high Tanimoto similarity (which may not necessarily equal 1 due to chemical structure variations and the presence of salts).

9. **2) It has been recognized that molecular docking on crystal structures isn't the best way forward, and I'm aware that real experts to a fair bit of preprocessing of the structures using e.g., long molecular dynamics simulations to identify pockets. Surely this would affect the authors' results.**

We thank the reviewer for this important comment. To our knowledge, long molecular dynamics simulations have been used in several studies that focus on a specific protein of interest to account for protein conformational changes. These may be important for the ligand binding activities of certain proteins like AcrB (Vargiu and Nikaido (2012) *PNAS* 109: 20637-20642 (2012); Weng et al (2021) *Sci. Rep.* 11: 7429; Kuzmanic et al (2020) *Acc. Chem. Res.* 53: 654-661). We acknowledge the fact that docking simulations with rigid proteins are more limited in this regard, and we view our docking as a baseline approach that is fully consistent with the rigid protein docking pipeline used in numerous docking studies, including benchmarking studies (Durrant et al, 2013; Pereira et al, 2016). In these studies, proteins have been assumed to be rigid, and their crystal structures have been directly used for docking. The advantage of this approach is that large-scale analyses are more computationally tractable, enabling both large-scale forward (Lyu et al. 2019; Bender et al. 2021) and reverse (Kharkar et al. 2014; Lee et al. 2016) docking applications. The disadvantage of this approach is that—as the reviewer suggests—situations in which protein conformational activity is important to ligand binding may not be accurately modeled. We believe that long molecular dynamics simulations require detailed, protein-specific information regarding interaction domains and key rate parameters, which fall beyond the scope of our large-scale study. To clarify this point, we have revised lines 429-434 to read: "Concomitantly, limitations to the development of more accurate docking methods are the use of rigid protein docking in this and other benchmarking studies (Durrant *et al*, 2013; Pereira *et al*, 2016) and the scarcity of benchmarking datasets. Long molecular dynamics simulations that focus on a specific protein of interest could account for protein conformational changes that, in certain cases like AcrB, might be important for ligand binding (Vargiu & Nikaido, 2012)." We think that these revisions better contextualize the limitations of our docking approach for all readers, and also make clear that what we have done—although coarse-grained—is consistent with prior docking studies and benchmarks.

We thank the reviewer again for their detailed and insightful comments, which have helped us to significantly improve our work.

Reviewer #3.

We thank the reviewer for their thoughtful and insightful report.

10. **In this paper, the authors sought to test whether AlphaFold2 protein structure predictions could be used for reverse docking, that is, prediction of binding targets of antibacterial compounds. They identify 218 compounds with anti-bacterial activity, which they seek to dock to 296 AlphaFold2-predicted essential protein structures. These predictions seemed to suggest widespread compound and protein promiscuity, which they experimentally validate using enzymatic inhibition assays. Using the results of these assays and published interactions, they however find that the accuracy of the reverse docking predictions is poor (auROC~0.5 on average). They do find that molecular docking using AlphaFold2-predicted structures provides similar performance compared to using experimentally determined structures. For AlphaFold2 structures, using machine learning-based scoring functions for docking in some cases improves the accuracy.**

We thank the reviewer for their interest and thoughtful suggestions, and we have revised the manuscript to address all the points raised.

11. **Overall this is an interesting idea but the conclusions are largely negative, and the main conclusion is that reverse docking as currently done provides poor performance. The contribution of AlphaFold2 predictions to this study is moderately clear.**

    We thank the reviewer for their interest and agree that our results suggest that further work in improving docking is needed to better leverage the protein structures predicted by AlphaFold. We feel that this point is especially timely in light of the excitement from AlphaFold and its implications for drug discovery. We would also point out that, while we have performed the first large-scale docking study involving AlphaFold2-predicted protein structures, we have not focused on evaluating the quality of AlphaFold structures in this work. Rather, we have aimed to determine whether a commonly used, reverse-docking approach to predicting protein-ligand interactions performs well given AlphaFold structures—a question which could inform our current abilities to make use of general structures given by AlphaFold. To this end, we hope that our work has provided a comprehensive and inspiring evaluation of different docking and scoring approaches, in addition to unique original datasets, that could determine whether we might be able to fully leverage AlphaFold protein structures in this way.

12. **One important technical aspect that is missing is confidence intervals for auROC. It is very hard to compare auROC values without such confidence intervals, and without them, one cannot conclude that one auROC value, eg obtained by ML based scoring functions is higher than another one. This needs to be addressed.**

    We thank the reviewer for this thoughtful comment. To address this point, we have provided 95% confidence intervals for all auROC (and area under the precision-recall curve, auPRC) values, as generated by bootstrapping (lines 871-873 in the *Methods*), in Table EV1. The calculated confidence intervals suggest that our values of the auROC (and auPRC) are robust to variability in the data; this is now mentioned on lines 281-283 and lines 338-341 of the revised paper. We believe that these confidence intervals also definitively show that the auROC values obtained by the machine learning-based rescoring fucntions are, for the most part, indeed higher than the corresponding AutoDock Vina baseline values (Table EV1).

    We thank the reviewer again for their thoughtful and insightful comments, which have helped us to significantly improve our work.

Thank you again for sending us your revised manuscript. We have now received the reports from two of the three reviewers who were asked to review four revised study. As you will see below, they are satisfied with the performed revisions and support publication. We have also been in contact with reviewer #2, who unfortunately did not have time to perform a full review, but did look at your point by point response and informally informed us that they do support publication. As such, I am pleased to inform you that your paper has been accepted for publication.

Reviewer #1:

The authors have addressed my prior comments.

Reviewer #3:

I remain somewhat skeptical about the contribution of this paper to the field except being the first paper to apply molecular docking to Alphafold2 structures. As the authors note, the performance of molecular docking in identifying true protein-ligand binding pairs is weak, whether applied to real structure or predicted structures. With all that said, the reviewers have addressed my few comments and other reviewers'. The paper is technically sound and I won't object to its publication.

EMBO Press Author Checklist

Corresponding Author Name: James J. Collins	
Journal Submitted to: Molecular Systems Biology	
Manuscript Number: MSB-2022-11081	

USEFUL LINKS FOR COMPLETING THIS FORM
The EMBO Journal - Author Guidelines
EMBO Reports - Author Guidelines
Molecular Systems Biology - Author Guidelines
EMBO Molecular Medicine - Author Guidelines

Reporting Checklist for Life Science Articles (updated January 2022)
This checklist is adapted from Materials Design Analysis Reporting (MDAR) Checklist for Authors. MDAR establishes a minimum set of requirements in transparent reporting in the life sciences (see Statement of Task: 10.31222/osf.io/9sm4x). Please follow the journal's guidelines in preparing your manuscript.
Please note that a copy of this checklist will be published alongside your article.

Abridged guidelines for figures
1. Data
The data shown in figures should satisfy the following conditions:
- the data were obtained and processed according to the field's best practice and are presented to reflect the results of the experiments in an accurate and unbiased manner.
- ideally, figure panels should include only measurements that are directly comparable to each other and obtained with the same assay.
- plots include clearly labeled error bars for independent experiments and sample sizes. Unless justified, error bars should not be shown for technical replicates.
- if n<5, the individual data points from each experiment should be plotted. Any statistical test employed should be justified.
- Source Data should be included to report the data underlying figures according to the guidelines set out in the authorship guidelines on Data Presentation.

2. Captions
Each figure caption should contain the following information, for each panel where they are relevant:
- a specification of the experimental system investigated (eg cell line, species name).
- the assay(s) and method(s) used to carry out the reported observations and measurements.
- an explicit mention of the biological and chemical entity(ies) that are being measured.
- an explicit mention of the biological and chemical entity(ies) that are altered/varied/perturbed in a controlled manner.
- the exact sample size (n) for each experimental group/condition, given as a number, not a range;
- a description of the sample collection allowing the reader to understand whether the samples represent technical or biological replicates (including how many animals, litters, cultures, etc.).
- a statement of how many times the experiment shown was independently replicated in the laboratory.
- definitions of statistical methods and measures:
  - common tests, such as t-test (please specify whether paired vs. unpaired), simple χ2 tests, Wilcoxon and Mann-Whitney tests, can be unambiguously identified by name only, but more complex techniques should be described in the methods section;
  - are tests one-sided or two-sided?
  - are there adjustments for multiple comparisons?
  - exact statistical test results, e.g., P values = x but not P values < x;
  - definition of 'center values' as median or average;
  - definition of error bars as s.d. or s.e.m.

Please complete ALL of the questions below.
Select "Not Applicable" only when the requested information is not relevant for your study.

Materials

Newly Created Materials	Information included in the manuscript?	In which section is the information available? (Reagents and Tools Table, Materials and Methods, Figures, Data Availability Section)
New materials and reagents need to be available; do any restrictions apply?	Not Applicable	

Antibodies	Information included in the manuscript?	In which section is the information available? (Reagents and Tools Table, Materials and Methods, Figures, Data Availability Section)
For **antibodies** provide the following information:<br>- Commercial antibodies: RRID (if possible) or supplier name, catalogue number and or/clone number<br>- Non-commercial: RRID or citation	Not Applicable	

DNA and RNA sequences	Information included in the manuscript?	In which section is the information available? (Reagents and Tools Table, Materials and Methods, Figures, Data Availability Section)
**Short novel DNA or RNA including primers, probes**: provide the sequences.	Not Applicable	

Cell materials	Information included in the manuscript?	In which section is the information available? (Reagents and Tools Table, Materials and Methods, Figures, Data Availability Section)
**Cell lines:** Provide species information, strain. Provide accession number in repository **OR** supplier name, catalog number, clone number, and/**OR** RRID.	Not Applicable	
**Primary cultures:** Provide species, strain, sex of origin, genetic modification status.	Not Applicable	
Report if the cell lines were recently **authenticated** (e.g., by STR profiling) and tested for mycoplasma contamination.	Not Applicable	

Experimental animals	Information included in the manuscript?	In which section is the information available? (Reagents and Tools Table, Materials and Methods, Figures, Data Availability Section)
**Laboratory animals or Model organisms:** Provide species, strain, sex, age, genetic modification status. Provide accession number in repository **OR** supplier name, catalog number, clone number, **OR** RRID.	Not Applicable	
**Animal observed in or captured from the field:** Provide species, sex, and age where possible.	Not Applicable	
Please detail **housing and husbandry conditions**.	Not Applicable	

Plants and microbes	Information included in the manuscript?	In which section is the information available? (Reagents and Tools Table, Materials and Methods, Figures, Data Availability Section)
**Plants:** provide species and strain, ecotype and cultivar where relevant, unique accession number if available, and source (including location for collected wild specimens).	Not Applicable	
**Microbes:** provide species and strain, unique accession number if available, and source.	Yes	The Materials and Methods section details microbial strains used.

Human research participants	Information included in the manuscript?	In which section is the information available? (Reagents and Tools Table, Materials and Methods, Figures, Data Availability Section)
If collected and within the bounds of privacy constraints report on age, sex and gender or ethnicity for all study participants.	Not Applicable	

Core facilities	Information included in the manuscript?	In which section is the information available? (Reagents and Tools Table, Materials and Methods, Figures, Data Availability Section)
If your work benefited from core facilities, was their service mentioned in the acknowledgments section?	Not Applicable	

Design

Study protocol	Information included in the manuscript?	In which section is the information available? (Reagents and Tools Table, Materials and Methods, Figures, Data Availability Section)
If study protocol has been **pre-registered, provide DOI in the manuscript**. For clinical trials, provide the trial registration number **OR** cite DOI.	Not Applicable	
Report the **clinical trial registration number** (at ClinicalTrials.gov or equivalent), where applicable.	Not Applicable	

Laboratory protocol	Information included in the manuscript?	In which section is the information available? (Reagents and Tools Table, Materials and Methods, Figures, Data Availability Section)
Provide DOI OR other citation details if **external detailed step-by-step protocols** are available.	Not Applicable	

Experimental study design and statistics	Information included in the manuscript?	In which section is the information available? (Reagents and Tools Table, Materials and Methods, Figures, Data Availability Section)
Include a statement about **sample size** estimate even if no statistical methods were used.	Yes	Materials and Methods and the Figure captions provide information regarding sample size, when relevant.
Were any steps taken to minimize the effects of subjective bias when allocating animals/samples to treatment (e.g. **randomization procedure**)? If yes, have they been described?	Not Applicable	
Include a statement about **blinding** even if no blinding was done.	Not Applicable	
Describe **inclusion/exclusion criteria** if samples or animals were excluded from the analysis. Were the criteria pre-established?<br><br>If sample or data points were omitted from analysis, report if this was due to attrition or intentional exclusion and provide justification.	Not Applicable	
For every figure, are **statistical tests** justified as appropriate? Do the data meet the assumptions of the tests (e.g., normal distribution)? Describe any methods used to assess it. Is there an estimate of variation within each group of data? Is the variance similar between the groups that are being statistically compared?	Not Applicable	

Sample definition and in-laboratory replication	Information included in the manuscript?	In which section is the information available? (Reagents and Tools Table, Materials and Methods, Figures, Data Availability Section)
In the figure legends: state number of times the experiment was **replicated** in laboratory.	Yes	Figures 3 and EV2 indicate experiments done in biological duplicate.
In the figure legends: define whether data describe **technical or biological replicates**.	Yes	Figures 3 and EV2 indicate experiments done in biological duplicate.

Ethics

Ethics	Information included in the manuscript?	In which section is the information available? (Reagents and Tools Table, Materials and Methods, Figures, Data Availability Section)
Studies involving **human participants**: State details of **authority granting ethics approval** (IRB or equivalent committee(s), provide reference number for approval.	Not Applicable	
Studies involving **human participants**: Include a statement confirming that **informed consent** was obtained from all subjects and that the experiments conformed to the principles set out in the WMA Declaration of Helsinki and the Department of Health and Human Services Belmont Report.	Not Applicable	
Studies involving **human participants:** For publication of **patient photos**, include a statement confirming that consent to publish was obtained.	Not Applicable	
Studies involving experimental **animals**: State details of **authority granting ethics approva**l (IRB or equivalent committee(s), provide reference number for approval. Include a statement of compliance with ethical regulations.	Not Applicable	
Studies involving **specimen and field samples:** State if relevant **permits** obtained, provide details of authority approving study; if none were required, explain why.	Not Applicable	

Dual Use Research of Concern (DURC)	Information included in the manuscript?	In which section is the information available? (Reagents and Tools Table, Materials and Methods, Figures, Data Availability Section)
Could your study fall under dual use research restrictions? Please check biosecurity documents and list of **select agents and toxins** (CDC): https://www.selectagents.gov/sat/list.htm	Not Applicable	
If you used a select agent, is the security level of the lab appropriate and reported in the manuscript?	Not Applicable	
If a study is subject to dual use research of concern regulations, is the name of the **authority granting approval and reference number** for the regulatory approval provided in the manuscript?	Not Applicable	

Reporting

The MDAR framework recommends adoption of discipline-specific guidelines, established and endorsed through community initiatives. Journals have their own policy about requiring specific guidelines and recommendations to complement MDAR.

Adherence to community standards	Information included in the manuscript?	In which section is the information available? (Reagents and Tools Table, Materials and Methods, Figures, Data Availability Section)
State if relevant guidelines or checklists (e.g., **ICMJE, MIBBI, ARRIVE, PRISMA**) have been followed or provided.	Not Applicable	
For **tumor marker prognostic studies,** we recommend that you follow the **REMARK** reporting guidelines (see link list at top right). See author guidelines, under 'Reporting Guidelines'. Please confirm you have followed these guidelines.	Not Applicable	
For **phase II and III randomized controlled trials**, please refer to the **CONSORT** flow diagram (see link list at top right) and submit the CONSORT checklist (see link list at top right) with your submission. See author guidelines, under 'Reporting Guidelines'. Please confirm you have submitted this list.	Not Applicable	

Data Availability

Data availability	Information included in the manuscript?	In which section is the information available? (Reagents and Tools Table, Materials and Methods, Figures, Data Availability Section)
Have **primary datasets** been deposited according to the journal's guidelines (see 'Data Deposition' section) and the respective accession numbers provided in the Data Availability Section?	Yes	All datasets generated are presented as Extended View Datasets.
Were **human clinical and genomic datasets** deposited in a public access-controlled repository in accordance to ethical obligations to the patients and to the applicable consent agreement?	Not Applicable	
Are **computational models** that are central and integral to a study available without restrictions in a machine-readable form? Were the relevant accession numbers or links provided?	Not Applicable	
If publicly available data were reused, provide the respective **data citations in the reference list**.	Yes	AlphaFold structures were obtained from the references cited.
